# Geographical, temporal, and individual‐based differences in the trophic ecology of female Cape fur seals

**DOI:** 10.1002/ece3.9790

**Published:** 2023-02-08

**Authors:** Jonathan A. Botha, Clive N. Trueman, Stephen P. Kirkman, John P. Y. Arnould, Amanda T. Lombard, Maëlle Connan, G. J. Greg Hofmeyr, S. Mduduzi Seakamela, Pierre A. Pistorius

**Affiliations:** ^1^ Marine Apex Predator Research Unit (MAPRU), Department of Zoology and Institute for Coastal and Marine Research Nelson Mandela University Gqeberha South Africa; ^2^ Ocean and Earth Science, Southampton University of Southampton Southampton UK; ^3^ Oceans and Coast Research, Department of Forestry Fisheries and the Environment Cape Town South Africa; ^4^ Faculty of Science, Engineering and Built Environment Deakin University Burwood Victoria Australia; ^5^ Port Elizabeth Museum at Bayworld Gqeberha South Africa

**Keywords:** *Arctocephalus pusillus pusillus*, foraging ecology, individual specialization, South Africa, stable isotope analysis

## Abstract

Information on resource use and trophic dynamics of marine predators is important for understanding their role in ecosystem functioning and predicting population‐level responses to environmental change. Where separate populations experience different local environmental conditions, geographic variability in their foraging ecology is often expected. Within populations, individuals also vary in morphology, physiology, and experience, resulting in specialization in resource use. In this context, isotopic compositions of incrementally grown tissues such as keratinous hairs offer a valuable opportunity to study long‐term variation in resource and habitat use. We investigated the trophic ecology of female Cape fur seals (*Arctocephalus pusillus pusillus*) using carbon and nitrogen isotopic compositions of serially sampled whiskers collected at four breeding sites along the coast of South Africa. Drawing on over 900 isotopic measurements, we assessed geographic variability in isotopic niche width between colonies and the degree of individual specialization. We found slight, but clear geographic differences in isotopic ratios and isotopic niche widths, seemingly related to ecological setting, with niche widths being proportional to the area of available shelf and shelf‐slope habitat surrounding the colony. We further identified periodic oscillations in isotopic ratios, which likely reflect temporal patterns in foraging distribution and prey type, linked to shifts in the availability of prey resources and their interaction with constraints on individual females throughout their breeding cycle. Finally, individual specialization indices revealed that each of the study populations contain specialist individuals that utilize only a small subset of the total population niche width. The degree of individual specialization was, however, not consistent across colonies and may reflect an interactive influence between density‐dependent effects and habitat heterogeneity. Overall, this study provides important information on the trophic ecology of Cape fur seals breeding in South Africa and highlights the need to consider geographic and individual variability when assessing the foraging ecology of marine predators.

## Graphical abstract

Using stable isotope compositions of serially sampled whiskers, this paper provides a detailed assessment of Cape fur seal trophic ecology across four South African breeding colonies. We focus on geographic differences in diet and habitat use, long‐term temporal trends in isotopic signatures, and geographic differences in individual specialization. Our results highlight clear geographical gradients in the isotopic composition of whiskers, which support recent evidence of spatial differences in habitat use and foraging strategies.

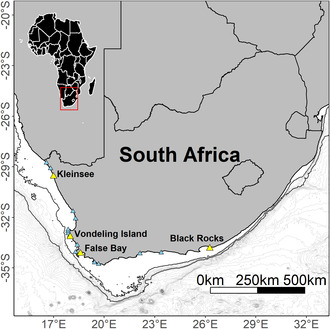

## 1 INTRODUCTION

The ecological niche has long been a central concept in understanding resource use of wild populations (reviewed in Leibold, 1995). Information on the ecological niche of a species contributes to a holistic understanding of ecosystem function to the benefit of conservation management and capabilities for predicting future trends in response to environmental variability (Augé et al., 2018; Hays et al., 2016, 2019; McGowan et al., 2016). In the marine environment, top predators play a crucial role in ecosystem structure and functioning, largely through their influence on prey populations (Roman et al., 2014; Young et al., 2015). Changes in the abundance and behavior of top predators can, therefore, be expected to have profound effects within marine ecosystems (Coleman & Williams, 2002; Heithaus et al., 2008). In addition, top predator ecology is linked to conditions at lower trophic levels and, as such, they are often considered as sentinels for monitoring ecosystem health and environmental change (Hazen et al., 2019; Hindell et al., 2003). Land breeding marine predators (e.g., seabirds and fur seals) may be especially useful sentinel species, as they are accessible at the breeding site during periods of central place foraging. In addition, they forage within a limited area during periods of offspring attendance and thus may be particularly sensitive to local environmental fluctuation (Fretwell & Trathan, 2019; Harding et al., 2007).

Understanding the role of top predators in terms of ecosystem functioning and predicting their response to changes requires a comprehensive assessment of foraging dynamics both between and within populations. Conspecifics from separate populations often experience different oceanographic conditions and ecological opportunity with regard to habitat availability and prey species (Newsome et al., 2015). In addition, the marine environment is often dynamic and the distribution and availability of resources can vary over multiple temporal scales (Hunt Jr et al., 2008; Weimerskirch, 2007). Consequently, patterns of resource‐ and habitat‐use of marine predators have often been shown to differ across geographic (Baylis et al., 2018; Drago et al., 2016; Handley et al., 2017) and temporal dimensions (Chambellant et al., 2013; Hume et al., 2004; Tarroux et al., 2018). Separate colonies may also experience different levels of density‐dependent competition, which could drive strategies of resource partitioning to facilitate coexistence (Corman et al., 2016; Newsome et al., 2015; Wakefield et al., 2013). Although this is often manifested through segregation between sex/age classes (Drago et al., 2015; Leung et al., 2012; Lewis et al., 2006; Newland et al., 2009), there is increasing evidence of individual‐level strategies within marine predator populations (Baylis et al., 2015; Bearhop et al., 2006; Camprasse, Cherel, Arnould, et al., 2017; Votier et al., 2017). Individuals within a single population are unlikely to be ecologically equivalent, and differences in their morphology, physiology, and experience are expected to impact foraging behaviorbehavio (Bolnick et al., 2003). Indeed, even populations of generalist species have been shown to include specialized individuals that utilize only a subset of the total available niche (Camprasse, Cherel, Bustamante, et al., 2017; Jaeger et al., 2010; Woo et al., 2008). Individual specialization may hold important fitness consequences and have far reaching implications with regard to the plasticity of a population in response to environmental change (Authier et al., 2012; Cucherousset et al., 2011). As such, it is important that individual‐specific behavior be accounted for in ecological studies.

Quantifying resource and habitat use over large spatiotemporal scales through traditional methods (diet assessments and biotelemetry) is labour‐intensive and costly. Assessing long‐term patterns at the individual level is especially challenging because this requires repeated sampling of the same individual across several years. In recent years, stable isotope analysis (SIA) of consumer tissues has increasingly gained popularity as a means to monitor the foraging ecology of upper trophic level species (Bearhop et al., 2004; Bolnick et al., 2002; Carneiro et al., 2017). Specifically, ratios of carbon stable isotopes (expressed as δ^13^C values) vary spatially within the marine environment and can be used as a proxy for the habitat from which prey species were acquired, and reflect the baseline isotopic signature (Cherel & Hobson, 2007; Trueman & St John Glew, 2019). In comparison, stable isotopes of nitrogen in animal tissues (expressed as δ^15^N values) increase in a stepwise manner with each trophic level, providing information on the trophic position of resources consumed (Minagawa & Wada, 1984). Assessed in conjunction, the isotopic composition of carbon and nitrogen can be used to define a population‐level isotopic niche, drawing ecological inference from distributions of individual isotope data plotted in cartesian space (Newsome et al., 2010).

The SIA of continuously growing, metabolically inert tissues provides a unique opportunity to assess long‐term temporal patterns in trophic ecology, given that compositions reflect trophic information at the time of deposition (Cardona et al., 2017). Therefore, by incrementally sampling these tissues, trophic information spanning a range of time scales can be obtained. For pinnipeds in particular, SIA of serially sampled whiskers has become a widely used method to identify long‐term trends in trophic ecology from the population to the individual level (Botta et al., 2018; Cherel et al., 2009; de Lima et al., 2019; Jones et al., 2020; Kernaléguen et al., 2012, 2016).

The Cape fur seal, *Arctocephalus pusillus pusillus*, is endemic to sub‐Saharan Africa, with a breeding distribution spanning from Baia dos Tigres in southern Angola, to Algoa Bay on the southeast coast of South Africa (Kirkman et al., 2013). The bulk of the population is concentrated within the Benguela Ecosystem, along the coastline of Namibia, and the west coast of South Africa. With population estimates numbering between 1.5 and 2 million individuals (Butterworth et al., 1995; Kirkman et al., 2007), the Cape fur seal comprises a major proportion of the southern African marine predator biomass and is a functionally important component of the Benguela marine ecosystem (Shannon et al., 2003). This ecosystem is characterized by high levels of temporal variability and is considered particularly vulnerable to climatic shifts (O'Toole et al., 2001; Pitcher et al., 1992). Indeed, changes in temperature and upwelling intensity have been apparent since the early 1990s (Jarre et al., 2015) and are thought to be at least partly responsible for the shifts in distribution and abundance of several important Cape fur seal prey species (Blamey et al., 2012; Roy et al., 2007). While this has had profound influences on the foraging behavior and diet of seabirds throughout the region (Crawford et al., 2014, 2016; Green et al., 2015), impacts on Cape fur seals are less well‐understood, and requires investigation at larger spatiotemporal scales.

Several previous studies have monitored the diet of Cape fur seals to determine diet composition and variability (Connan et al., 2014; de Bruyn et al., 2003, 2005; Huisamen et al., 2012; Mecenero, Roux, Underhill, Bester, & Kirkman, 2006), or to provide consumption estimates (David, 1987; Mecenero, Kirkman, & Roux, 2006; Punt & Butterworth, 1995). Many such studies have been motivated by the requirement to assess perceived competition between the fur seals and commercial fisheries (David, 1987; Mecenero et al., 2007; Shaughnessy, 1985; Wickens et al., 1992; Wickens & Sims, 1994). In addition, the need for enhanced monitoring and establishing of baselines has been motivated by requirements for measuring ecosystem change in the Benguela (Kirkman, 2007; Kirkman et al., 2011), including detailed information on the foraging behavior and habitat use of Cape fur seals. However, compared with other otariid species (e.g., Arnould & Hindell, 2001; de Bruyn et al., 2009; Guinet et al., 2001; Harcourt et al., 2002; Luque et al., 2007), many of these fundamental aspects have remained poorly investigated in Cape fur seals (Botha et al., 2020; Kirkman et al., 2019).

A thorough assessment of geographic, temporal, and individual patterns in the habitat and resource use of Cape fur seals is relevant and timely for several reasons. Although the diet of Cape fur seals has been reported for several South African colonies and over various temporal scales (Connan et al., 2014; Huisamen et al., 2012), comparative assessments across breeding sites are limited to the Namibian population (de Bruyn et al., 2003, 2005; Mecenero, Roux, Underhill, & Bester, 2006). Similar spatiotemporal investigations are required for the South African population, especially considering that these breeding colonies are subject to vastly different oceanographic regimes (Hutchings et al., 2009; Kirkman et al., 2016). In addition, while previous studies suggest that female Cape fur seals forage mainly over the continental shelf and shelf‐slope (Botha et al., 2020; Skern‐Mauritzen et al., 2009), much of this information is limited to the lactation phase during which females are central‐place foragers (David & Rand, 1986). It is possible that outside of these periods, females may differ in their spatial distribution and habitat use (Beauplet et al., 2004; Costa & Gales, 2003). Finally, while Cape fur seals are typically regarded as generalist foragers, a recent study highlighted intracolony differences in foraging strategies, whereby individuals vary in the degree of pelagic and benthic diving behavior (Kirkman et al., 2019). It is not yet known whether these differences translate into individual specialization because an understanding of this requires longitudinal sampling of specific individuals over extended periods. By applying SIA to serially sampled whiskers, this study aimed to assess the trophic ecology of female Cape fur seals from four South African breeding colonies. Specifically, the objectives were to: (1) investigate inter‐ and intracolony differences in isotopic composition and isotopic niche width; (2) determine whether Cape fur seals show periodic oscillations in isotopic signatures which may be attributed to annual changes; and (3) investigate the level of individual specialization in resource use within and among colonies.

## 2 MATERIALS AND METHODS

### 2.1 Ethics statement

All data collection was conducted under approval by the Animal Ethics Committee of the Department of Forestry, Fisheries, and the Environment, then known as the Department of Environmental Affairs (Ref: EC‐2015‐5), and the Research Ethics Committee at the Nelson Mandela University, then known as the Nelson Mandela Metropolitan University (reference: A13‐SCI‐ZOO‐008).

### 2.2 Data collection and stable isotope analysis

**FIGURE 1:**
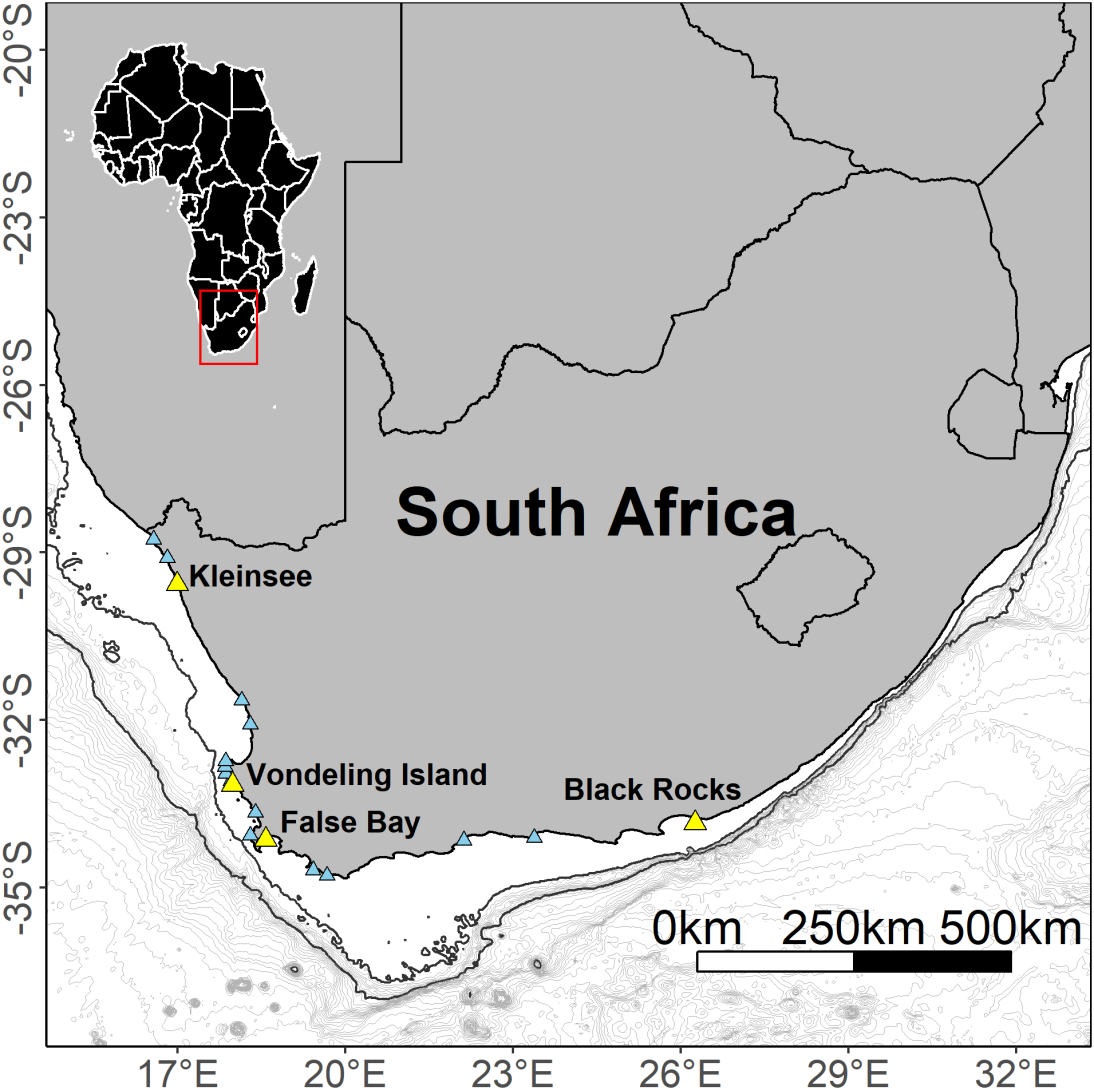
Distribution of Cape fur seal breeding colonies in South Africa (blue triangles), including the four study sites (yellow triangles). Bathymetry is presented at 100 m intervals between the 200 m and 1000 m isobaths (black lines). Beyond the 1000 m isobath, bathymetry is presented at 200 m intervals.

Sample collection was carried out at four South African Cape fur seal breeding colonies, namely Kleinsee, Vondeling Island, Seal Island (False Bay), and Black Rocks (Algoa Bay) during the austral winters of 2014 and 2015 (Figure 1). These four colonies differ substantially in population size with the mainland colony at Kleinsee being the largest (annual pup production (APP): 50 000–80 000), followed by Vondeling Island (APP: 17 000–23 000), False Bay (APP: 14 000–19 000), and Black Rocks (APP: 90–1700) (Kirkman et al., 2007). At each site, adult females suckling pups were captured using a modified hoop net (David et al., 1990). At Kleinsee, Vondeling Island and False Bay, individuals were anaesthetized using a portable vaporizer (Stinger, Advanced Anaesthesia Specialists, Gladesville, New South Wales, Australia) (Gales & Mattlin, 1998). Once anaesthetized, the animal was removed from the net and placed on a rectangular wooden board, to provide a flat surface on which to work. At Black Rocks, captured individuals were restrained within the hoop net during data collection procedures. At all sites, seals were captured primarily for the deployment of tracking and behavioral recording devices (see Botha et al., 2020). In addition, and for the purpose of this study, one to two whiskers were collected from each individual seal, using a pair of scissors to clip the whisker as close to the skin as possible. Following data collection, individuals were released back into the colony and monitored until normal behaviors resumed.

Sample processing and stable isotope analysis were conducted at the School of Ocean and Earth Science, University of Southampton, UK. Prior to isotopic analyses, whiskers were hand‐washed with 100% ethanol and cleaned using distilled water. They were then dried, measured, and sectioned into 3‐mm‐long segments from the proximal (facial) to the distal end (tip). Each section was weighed on a microbalance, and then subsampled by cutting longitudinally, to produce samples in a mass range of (0.3–1.99 mg) and packaged into tin containers. Carbon and nitrogen isotope analysis was performed simultaneously via continuous‐flow isotope ratio mass spectrometry using a Vario Isotope select elemental analyzer, coupled to an Isoprime 100 isotope mass spectrometer. Replicates using internal laboratory standards (L‐glutamic acid (C), Glutamic acid (CT standard), acetanilide and protein standard OAS) were used for quality control and calibration. Long‐term analytical precision assessed from internal standards was better than 0.2‰ for both isotopes. The average C:N ratios for individuals ranged from 2.6 to 2.8, within the acceptable theoretical range for pure keratin (3.4 ± 0.5, O'Connell & Hedges, 1999). In six of the samples, C:N ratios were comparatively low (C:N <2). However, isotopic values of carbon and nitrogen for these six samples did not vary substantially from the remaining samples, and exclusion of these samples did not significantly alter results. As such, they were retained for all further analysis to maintain the temporal consistency across individuals.

Isotopic ratios (*R*) of carbon and nitrogen were expressed as delta values:

$$\delta X=\left(\frac{R_{\text{sample}}}{R_{\text{standard}}}\right)-1$$

where *X* is ^13^C or ^15^N, *R*_sample_ and *R*_standard_ represent the isotopic ratios of the samples and standards (Vienna PeeDee Belemnite and atmospheric N2), respectively.

### 2.3 Statistical analysis

Data processing and statistical analyses were performed in the R statistical environment, version 3.5.1 (R Core Team, 2020). Intercolony differences in whisker δ^13^C and δ^15^N values were assessed within a linear mixed‐effects modelling framework using the package *lme4* (Bates et al., 2015). Isotopic values of each whisker sample were modelled in response to colony as a fixed effect, and seal ID as a random effect to account for repeated measures per individuals. To estimate the variance explained by both the fixed‐ and random‐effects, a pseudo‐*R* ^2^ value was calculated for each model (Nakagawa & Schielzeth, 2013). Models were validated using quantile–quantile plots to assess normality, and residuals were plotted against fitted values to assess homogeneity.

Inter‐ and intracolony differences in isotopic niche area and overlap were assessed using Stable Isotope Bayesian Ellipses fitted through the *SIBER* package (Jackson et al., 2011). For each colony and individual, standard ellipse areas corrected for small sample size (SEA_C_) were determined and used to compare isotopic niche width and overlap. SEA_C_s was set to contain approximately 40% of the data and are increasingly being used as a measure of the core isotopic niche for marine predators (e.g. Botta et al., 2018; Dimitrijević et al., 2018; Handley et al., 2017). To estimate uncertainty and statistically compare ellipse areas between colony and individual groupings, Bayesian estimates of the standard ellipse area (SEA_B_) were calculated based on 10,000 simulated posterior ellipses. Posterior distributions and overlap of the 95% credibility intervals for SEA_B_s were assessed by means of density plots and probability estimates for differences in isotopic niche width between groups were computed. The degree of isotopic niche overlap between each group (colony and individuals) was calculated as a proportion of the overlapping area between the two SEA_C_ ellipses.

Vibrissal growth rates in wild seal populations have only been determined for South American fur seals, *Arctocephalus australis* (de Lima et al., 2019). However, periodic oscillations of isotopic ratios along the length of the whisker have previously been identified in several otariid species (Cherel et al., 2009; Kernaléguen et al., 2012, 2016). With the assumption that these cycles are annual, growth rates have subsequently been estimated. To assess whether such periodic oscillations occur along the whiskers of Cape fur seals, a wavelet analysis was performed independently on the δ^13^C and δ^15^N values along the length of each whisker using the package *WaveletComp* (Roesch & Schmidbauer, 2018). This information was then used to estimate growth rates for each individual. Given that whiskers were cut and not plucked, sections underneath the skin that contain the most recent trophic information, were not available. Therefore, a time‐synchronization was performed on the δ^13^C values following the methods by Kernaléguen et al. (2012), by means of a cross‐correlation analysis using the package *quantmod* (Ryan et al., 2020). Cross‐correlations were performed separately for each colony, and time‐series to a reference whisker, selected as the one with the highest number of significant cross‐correlations. Whiskers collected from females at the Black Rocks colony were substantially shorter (16–55 mm) compared with those collected from the three remaining colonies (79–220 mm) (Table 1). As such, it was not possible to identify patterns across meaningful temporal scales for the Black Rocks animals and, thus, these data were excluded from the temporal and individual specialization analyses.

**TABLE 1:** Summary information for whiskers sampled from female Cape fur seals at Kleinsee, Vondeling Island, False Bay and Black Rocks.

Seal ID	Colony	Year	Whisker length (mm)	No. samples	δ^13^C (‰)		δ^15^N (‰)	
					Mean ± SE	Range	Mean ± SE	Range
Seal 1	Kleinsee	2015	176.1	53	−13.6 ± 0.04	−14.4 to −12.9	15.9 ± 0.04	15.3 to 16.5
Seal 2	Kleinsee	2015	194.0	61	−13.4 ± 0.05	−14.2 to −12.5	15.9 ± 0.04	15.2 to 16.5
Seal 3	Kleinsee	2015	152.0	50	−13.3 ± 0.04	−14.0 to −12.9	16.4 ± 0.04	15.8 to 17.0
Seal 4	Kleinsee	2015	145.5	48	−13.5 ± 0.03	−14.0 to −13.0	16.2 ± 0.04	15.6 to 16.9
Seal 5	Kleinsee	2015	220.0	67	−13.2 ± 0.03	−14.1 to −12.7	16.7 ± 0.04	15.4 to 17.4
Seal 6	Kleinsee	2015	158.0	52	−13.4 ± 0.04	−14.1 to −12.9	15.9 ± 0.05	15.2 to 16.7
Seal 7	Kleinsee	2015	110.0	36	−13.7 ± 0.04	−14.3 to −13.2	16.0 ± 0.05	15.2 to 16.6
Seal 8	Kleinsee	2015	115.8	37	−13.7 ± 0.03	−14.2 to −13.3	16.0 ± 0.05	15.5 to 16.8
Seal 9	Kleinsee	2015	175.0	58	−13.4 ± 0.03	−13.9 to −13.1	16.7 ± 0.04	15.7 to 17.2
Seal 10	Kleinsee	2015	130.0	43	−13.4 ± 0.03	−13.8 to −13.0	16.0 ± 0.05	15.3 to 16.7
Seal 11	Vondeling Island	2014	144.5	42	−13.3 ± 0.06	−13.9 to −12.7	16.0 ± 0.06	15.3 to 16.8
Seal 12	Vondeling Island	2014	80.5	24	−13.4 ± 0.08	−14.2 to −12.7	15.7 ± 0.06	15.2 to 16.2
Seal 13	Vondeling Island	2014	109.5	33	−12.9 ± 0.07	−13.8 to −12.4	15.7 ± 0.05	15.2 to 16.6
Seal 14	Vondeling Island	2014	78.9	26	−13.3 ± 0.07	−13.9 to −12.6	15.7 ± 0.05	15.1 to 16.2
Seal 15	Vondeling Island	2014	106.9	35	−13.3 ± 0.07	−14.1 to −12.6	15.7 ± 0.06	15.1 to 16.6
Seal 16	False Bay	2015	148.0	51	−13.2 ± 0.05	−13.9 to −12.7	15.4 ± 0.04	14.3 to 15.9
Seal 17	False Bay	2015	97.0	31	−13.3 ± 0.04	−13.8 to −12.9	15.6 ± 0.08	14.7 to 16.4
Seal 18	False Bay	2015	95.0	31	−12.9 ± 0.06	−13.6 to −12.5	15.7 ± 0.05	15.1 to 16.1
Seal 19	False Bay	2015	123.0	41	−13.0 ± 0.05	−13.7 to −12.3	15.3 ± 0.07	14.6 to 16.5
Seal 20	False Bay	2015	86.9	27	−13.2 ± 0.05	−13.7 to −12.7	16.4 ± 0.07	15.7 to 16.9
Seal 21	False Bay	2015	128.2	38	−13.0 ± 0.05	−13.8 to −12.6	15.8 ± 0.05	15.1 to 16.4
Seal 22	Black Rocks	2014	20.0	6	−13.0 ± 0.04	−13.2 to −12.9	15.9 ± 0.1	15.6 to 16.2
Seal 23	Black Rocks	2014	55.2	18	−12.9 ± 0.03	−13.2 to −12.7	16.0 ± 0.06	15.5 to 16.5
Seal 24	Black Rocks	2014	43.7	13	−13.4 ± 0.04	−13.8 to −13.2	15.5 ± 0.05	15.3 to 15.8
Seal 25	Black Rocks	2014	16.0	5	−12.9 ± 0.04	−13.0 to −12.8	15.9 ± 0.12	15.5 to 16.1

The degree of individual specialization for each colony was calculated separately for δ^13^C and δ^15^N using Roughgarden's WIC/TNW index implemented with the package *RInSp* (Zaccarelli et al., 2013). Accordingly, the Total Niche Width (TNW) of a population comprises a Between‐Individual Component (BIC) and a Within‐Individual Component (WIC) (Bolnick et al., 2003). Determining the ratio WIC/TNW thus provides a good indication of the degree of individual specialization in a given population, with values closer to 1 being characteristic of generalist populations and values closer to 0 being characteristic of populations composed of more specialized individuals (Roughgarden, 1974). For each colony, WIC/TNW indices of δ^13^C and δ^15^N were calculated for both the first 24 segments (common to all individuals), and for all segments available. A non‐parametric Monte Carlo bootstrap technique was adopted to test the significance of the WIC/TNW ratio, by which 1000 replicates were generated to test the null hypothesis that all individuals were generalists. Unless stated otherwise, results are presented as mean ± standard error (SE).

## 3 RESULTS

Whiskers from 25 female Cape fur seals were analyzed (Table 1). Individual whisker lengths ranged from 16 to 220 mm (116.0 ± 10.2 mm) and carbon and nitrogen stable isotope ratios were obtained for 926 individual fragments, with the number of analyzed fragments ranging from five to 67 per individual (37.0 ± 3.3, Table 1). Whisker δ^13^C values ranged from −14.4‰ to −12.3‰ while δ^15^N values ranged from 14.2‰ to 17.4‰.

### 3.1 Inter and intra‐colony differences

**FIGURE 2:**
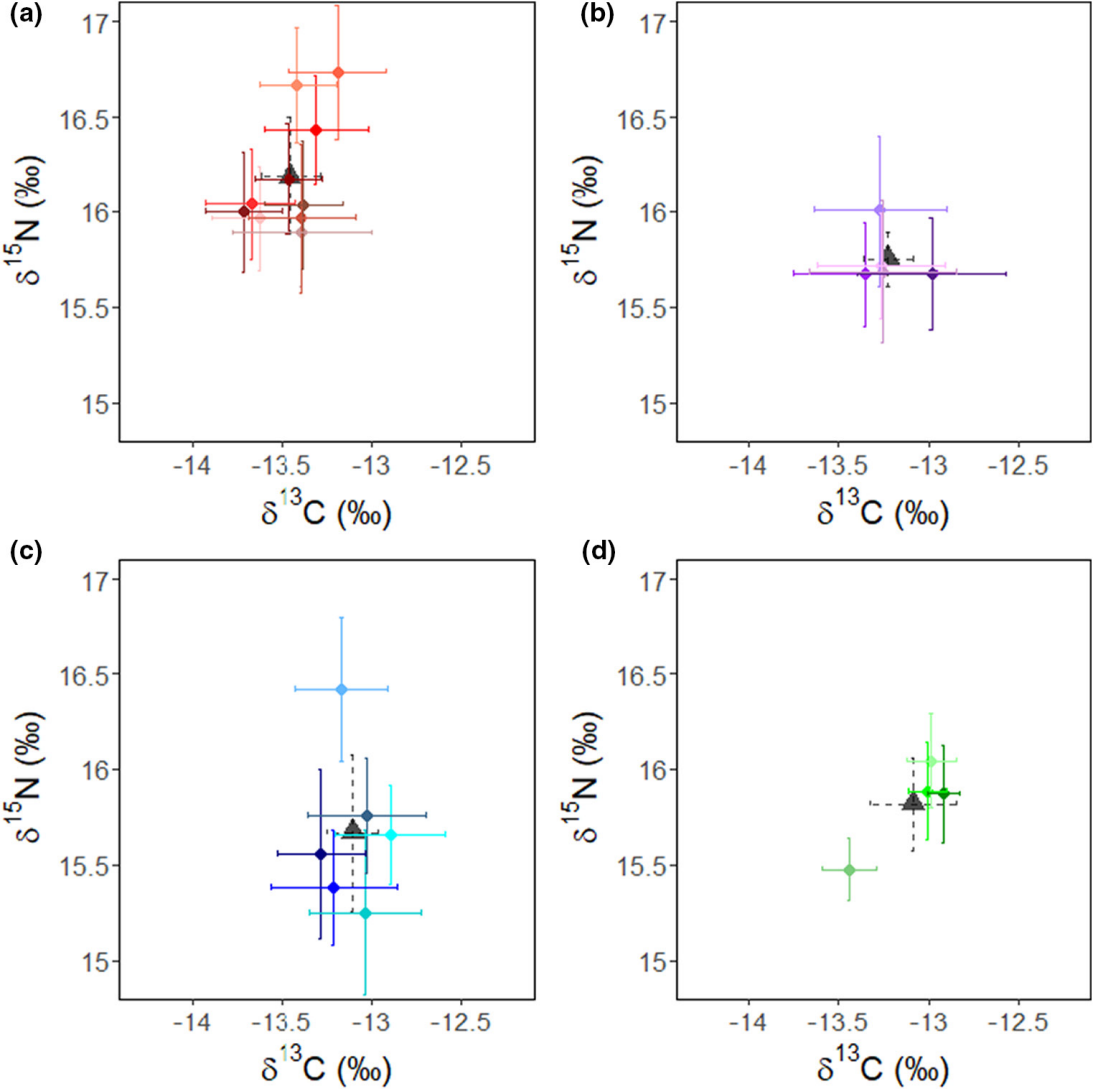
Stable isotope bi‐plots indicating the mean ± SE of δ^13^C and δ^15^N values of female Cape fur seals from Kleinsee (a), Vondeling Island (b), False Bay (c), and Black Rocks (d). Information for each individual is presented as a unique colour and the mean ± SE for each colony is indicated by the grey triangle and dashed lines.

Linear mixed‐effects models (LMEs) revealed slight, but clear differences in both δ^13^C and δ^15^N between colonies (Table 2). For δ^13^C, values typically increased from west to east with individuals at Black Rocks exhibiting similar values to individuals from False Bay, slightly higher average values than individuals from Vondeling Island (0.15‰), and higher values than individuals at Kleinsee (0.4 ‰) (Figure 2). By contrast, δ^15^N values did not display the same west to east gradient and were, on average, highest for individuals from Kleinsee and lowest for individuals from False Bay (Figure 2). In addition, while δ^15^N values for Kleinsee individuals were significantly higher than individuals from Vondeling Island (0.4‰) and False Bay (0.6‰), differences between Kleinsee and Black Rocks were not significant (Table 2). Pseudo‐*R* ^2^ values calculated for LMEs indicated a 17% and 35 % increase in the proportion of variance explained with the inclusion of fixed and random effects for δ^13^C and δ^15^N values, respectively. This suggests a moderate level of interindividual variability in isotopic compositions within colonies and is supported by comparisons of mean isotopic values between individuals at each colony (Figure 2).

**TABLE 2:** Results of the linear mixed effects models for the effect of colony on whisker δ^13^C and δ^15^N values for female Cape fur seals.

Response	Predictor (Colony)	CE (SE)	Df	*t* value	*p*‐value	*R* ^2^m	*R* ^2^c
δ^13^C	Intercept (Kleinsee)	−13.5 (0.1)	18.7	−261.9	**<.001**	0.18	0.35
	Vondeling Island	0.3 (0.1)	19.7	2.5	**.02**		
	False Bay	0.4 (0.1)	19.3	4.1	**<.001**		
	Black Rocks	0.4 (0.1)	27.5	3.3	**.002**		
δ^15^N	Intercept (Kleinsee)	16.2 (0.1)	20.6	167.5	**<.001**	0.23	0.58
	Vondeling Island	−0.4 (0.2)	20.9	−2.6	**.02**		
	False Bay	−0.5 (0.2)	20.9	−3.3	**.003**		
	Black Rocks	−0.4 (0.2)	23.9	−1.9	0.06		

*Note*: Significant *p*‐values (*p* < 0.05) are highlighted in bold.

**FIGURE 3:**
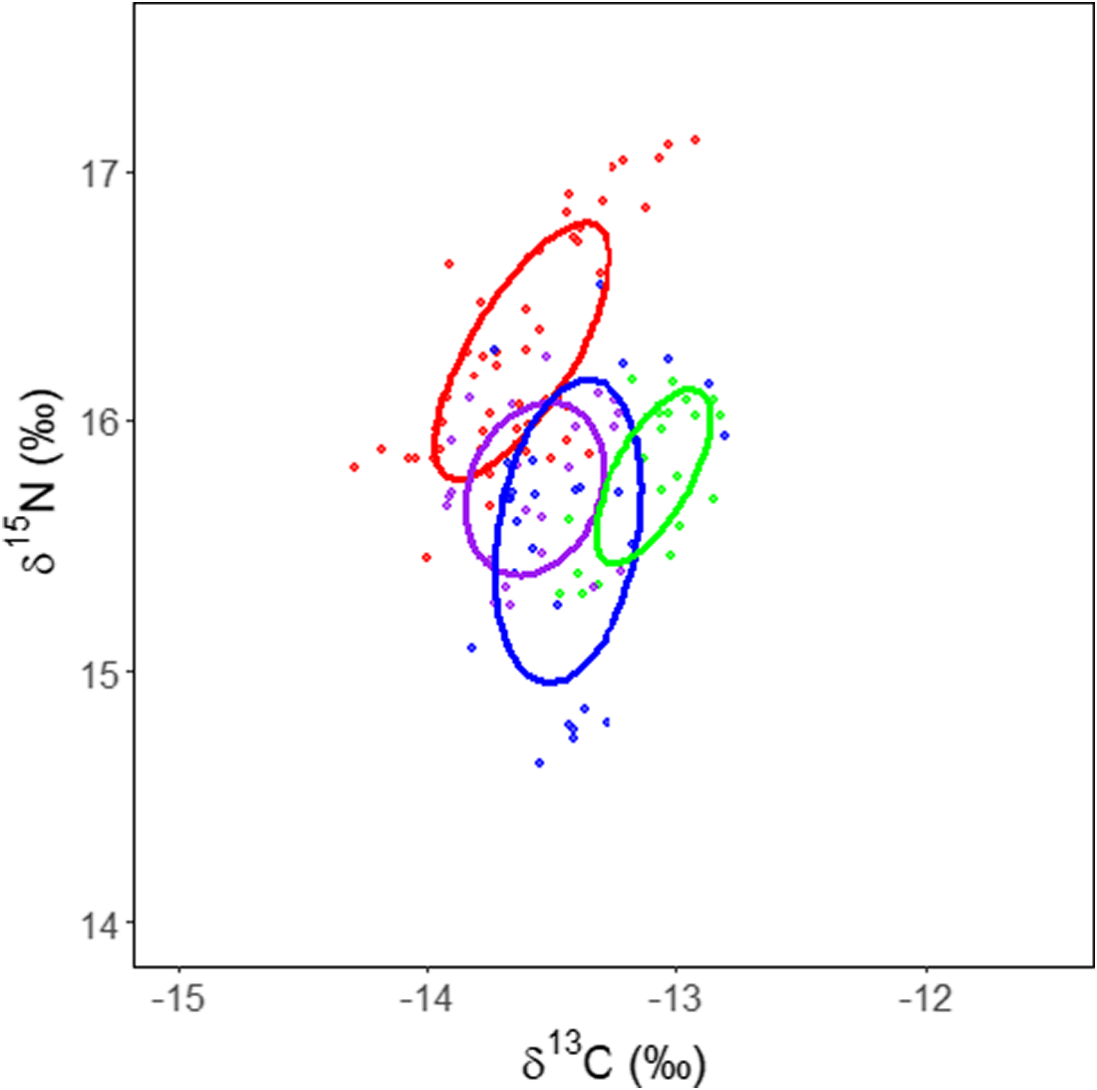
Standard Ellipse Areas corrected for small sample size (SEAc) set to include 40 % of the data, calculated based on the first five whisker fragments of female Cape fur seals for Kleinsee (red), Vondeling Island (purple), False Bay (blue) and Black Rocks (green). Points indicate the δ^13^C and δ^15^N for each 3 mm whisker fragment.

Comparison of the isotopic niche area based on the first five whisker fragments (common to all whiskers) showed that the False Bay grouping occupied the largest area (SEA_C_ = 0.4‰, SEA_B_ = 0.4 (0.1–0.2)). Probabilistic analysis based on Bayesian estimation further revealed that the isotopic niche width for False Bay was similar to the Kleinsee grouping (SEA_C_ = 0.3‰, SEA_B_ = 0.3 (0.2–0.4), *p* = .08), but was substantially larger than the Vondeling Island (SEA_C_ = 0.2 ‰, SEA_B_ = 0.2 (0.1–0.3), *p* = .01) and Black Rocks groupings (SEA_C_ = 0.2 ‰, SEA_B_ = 0.1 (0.1–0.2), *p* < .001). Furthermore, the isotopic niche area for Black Rocks was substantially smaller than the Kleinsee grouping (*p* < .01) but similar to the Vondeling Island group (*p* = .09). Kleinsee and Vondeling Island, however, occupied a similar sized isotopic niche (*p* = .13). The largest isotopic niche overlap was between Vondeling Island and False Bay, with the SEA_C_ overlapping by 30% (Figure 3). The isotopic niche for Kleinsee was more distinct from the other colonies with a 1.1% overlap with Vondeling Island, <1% overlap with False Bay and no overlap with Black Rocks (Figure 3). Furthermore, the Black Rocks grouping overlapped only 6.7% with the False Bay group and showed no overlap with the Vondeling Island grouping (Figure 3).

**FIGURE 4:**
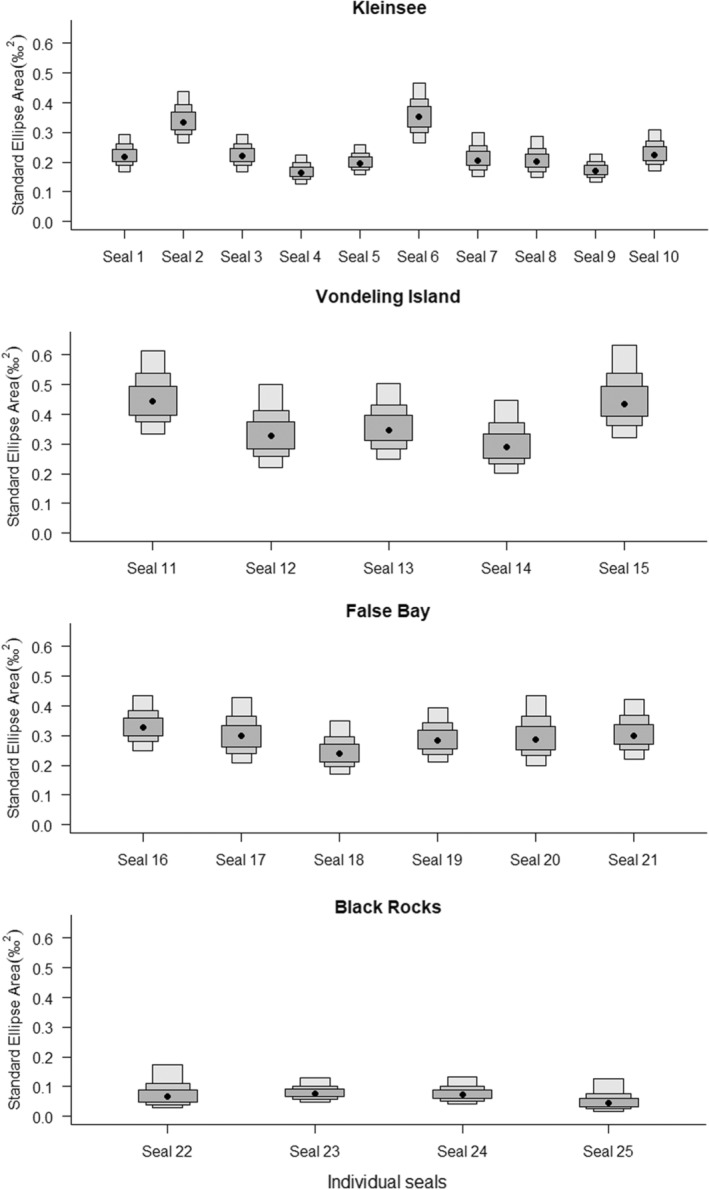
Bayesian standard ellipse areas (SEA_B_) estimated for the whiskers δ^13^C and δ^15^N values of female Cape fur seals from Kleinsee, Vondeling Island, False Bay, and Black Rocks.

Within colonies, individual variability in trophic niche width was typically low with the only notable differences recorded between individuals from Kleinsee, where two individuals occupied significantly larger isotopic areas than the rest (Table 3 and Figure 4). Isotopic overlap among individuals was generally higher for animals from Vondeling Island (39.5 ± 5.8 %, 14.4%–67.9%) compared with other colonies. The degree of overlap was more variable between individuals from both Kleinsee (18.2% ± 3.0%, 0%–74%) and False Bay (13.5% ± 4.1%, 0–49.8 %) (Table 3, Figure A1). Individuals from the Black Rocks colony had a moderate degree of overlap (9.0% ± 5.6%, 0%–32 %), except for a single individual that occupied an isotopic area separate from the three remaining animals (Table 3, Figure A1).

**TABLE 3:** Summary information for the Stable Isotope Bayesian Ellipse analysis conducted on whisker δ^13^C and δ^15^N values of female Cape fur seals from Kleinsee, Vondeling Island, False Bay and Black Rocks.

		SEA_C_ (‰^2^)	SEA_B_ (95 CI) (‰^2^)	SEA_C_ Overlap (%)	
Seal ID	Colony			Mean ± SE	Range
Seal 1	Kleinsee	0.23	0.22 (0.17–0.29)	26.01 ± 7.7	<1–61.15
Seal 2	Kleinsee	0.35	0.35 (0.27–0.44)	21.58 ± 7.02	0–61.71
Seal 3	Kleinsee	0.23	0.22 (0.17–0.30)	11.1 ± 3.26	0.47–26.47
Seal 4	Kleinsee	0.17	0.17 (0.13–0.22)	21.26 ± 4.34	2.62–42.26
Seal 5	Kleinsee	0.20	0.20 (0.16–0.26)	5.5 ± 3.41	0–24.04
Seal 6	Kleinsee	0.36	0.35 (0.27–0.47)	24.53 ± 7.85	<1–61.85
Seal 7	Kleinsee	0.22	0.22 (0.15–0.30)	24.28 ± 8.8	0–74.26
Seal 8	Kleinsee	0.21	0.20 (0.15–0.29)	20.53 ± 9.02	0–74.26
Seal 9	Kleinsee	0.18	0.17 (0.14–0.23)	4.68 ± 2.87	0–22.87
Seal 10	Kleinsee	0.24	0.23 (0.17–0.31)	22.5 ± 7.16	<1–61.85
Seal 11	Vondeling Island	0.47	0.45 (0.33–0.61)	25.25 ± 3.56	14.35–31.2
Seal 12	Vondeling Island	0.35	0.32 (0.22–0.50)	45.89 ± 10.18	24.31–67.94
Seal 13	Vondeling Island	0.37	0.35 (0.25–0.50)	28.87 ± 4.66	14.35–37.06
Seal 14	Vondeling Island	0.32	0.30 (0.20–0.44)	49.26 ± 8.26	31.2–67.94
Seal 15	Vondeling Island	0.47	0.44 (0.32–0.63)	48.32 ± 7.43	31.12–62.98
Seal 16	False Bay	0.34	0.32 (0.25–0.43)	18.01 ± 7.05	<1–42.49
Seal 17	False Bay	0.31	0.30 (0.21–0.43)	16.14 ± 7.38	<1–42.49
Seal 18	False Bay	0.25	0.24 (0.17–0.35)	16.75 ± 7.88	<1–49.77
Seal 19	False Bay	0.29	0.28 (0.21–0.39)	11.07 ± 4.79	<1–28.86
Seal 20	False Bay	0.31	0.28 (0.20–0.44)	0.01 ± 0.01	<1–0.06
Seal 21	False Bay	0.32	0.30 (0.22–0.42)	15.95 ± 7.84	0–49.77
Seal 22	Black Rocks	0.09	0.06 (0.03–0.17)	17.41 ± 8.1	0–32.05
Seal 23	Black Rocks	0.08	0.08 (0.05–0.13)	12 ± 8.74	0–32.05
Seal 24	Black Rocks	0.08	0.07 (0.04–0.13)	0 ± 0	0–0
Seal 25	Black Rocks	0.05	0.04 (0.02–0.13)	8.04 ± 5.35	0–20.18

*Note*: Standard ellipse area corrected for small sample size (SEA_C_) set to include 40 % of the data as well as modal values for Bayesian estimates of the standard ellipse area (SEA_B_) are provided. Overlap of the standard ellipse area for seals from the same colony is expressed as Mean ± SE and Range.

### 3.2 Periodicity in isotopic signatures

Wavelet analysis identified significant periodicity in the δ^13^C and δ^15^N values of all analyzed whiskers (Figure A2). Periodic cycles were detected every seven to 13 segments (21 to 39 mm), although this varied between individuals (Table 4). In 18 of the 21 whiskers, cycles occurred at similar periods for δ^13^C and δ^15^N values. Assuming that these cycles were annual, growth rates of female Cape fur seal whiskers in this study ranged from 0.06 to 0.1 mm.day^−1^, with each 3 mm segment, on average, representing an integration period of 38.9 ± 1.3 days. A total of 45, 10 and 15 cross‐correlations were performed on the δ^13^C time series from Kleinsee, Vondeling Island, and False Bay, respectively. This resulted in significant cross‐correlations in nine of the 21 whiskers (Kleinsee:3, Vondeling Island: 2, False Bay: 4), for which the time series were then adjusted to that of the reference whisker. The time series of the 12 remaining whiskers, for which there were no significant cross‐correlations, were left unchanged. On average, whiskers represented isotopic information spanning a period of 4.5 ± 0.3 y (range: 2.6–7.5 y).

**TABLE 4:** Periodicity results obtained from the Wavelet analysis conducted on whiskers collected from individual female Cape fur seals at Kleinsee, Vondeling Island, and False Bay.

	Kleinsee (*n* = 10)	Vondeling Island (*n* = 5)	False Bay (*n* = 6)
Whisker length (mm)	157.64 ± 10.96	104.06 ± 11.96	113.02 ± 9.69
Periodicity δ^13^C time series (mm)	29.97 ± 1.23	28.95 ± 2.58	26.66 ± 1.90
Periodicity δ^15^N time series (mm)	32.81 ± 1.84	29.44 ± 2.12	27.09 ± 2.35
No. of cycles	5.33 ± 0.41	3.61 ± 0.34	4.39 ± 0.56
Growth rate (mm.day^−1^)	0.08 ± 0.00	0.08 ± 0.01	0.07 ± 0.00

### 3.3 Individual specialization

Across all individuals, isotopic variability along the whisker length was always greater for the δ^15^N values than for the δ^13^C values. At all colonies, individual specialization indices (WIC/TNW) were slightly higher for both δ^13^C and δ^15^N values, when calculated using the full whisker compared with the first 24 fragments (Table 5). Simulations based on Monte Carlo bootstrapping, however, identified a high level of significance for all WIC/TNW ratios. As such, the null hypothesis specifying that all individuals are generalists was rejected, suggesting that individual specialization was present across all breeding sites. All colonies typically returned high WIC/TNW ratios for δ^13^C, although Kleinsee had a slightly lower ratio compared with Vondeling Island and False Bay (Table 5). For δ^15^N, WIC/TNW ratios were substantially lower for the Kleinsee and False Bay colonies, compared with the Vondeling Island colony (Table 5).

**TABLE 5:** Measure of individual specialization of female Cape fur seals from Kleinsee, Vondeling Island and False Bay as shown by total total niche width of the population (TNW), intra‐individual variability (WIC), inter‐individual variability (BIC), and the WIC/TNW index as a measure of individual specialization.

	Full whisker						First 24 fragments				
		TNW	WIC	BIC	WIC/TNW	*p*‐value	TNW	WIC	BIC	WIC/TNW	*p*‐value
δ^13^C	Kleinsee	0.09	0.07	0.03	0.73	**.001**	0.08	0.05	0.04	0.57	**.001**
	Vondeling Is.	0.16	0.15	0.02	0.9	**.006**	0.16	0.14	0.02	0.87	**.003**
	False Bay	0.11	0.09	0.02	0.84	**.001**	0.11	0.09	0.02	0.79	**.001**
δ^15^N	Kleinsee	0.18	0.1	0.09	0.53	**.001**	0.19	0.07	0.12	0.39	**.001**
	Vondeling Is.	0.12	0.1	0.02	0.86	**.001**	0.12	0.1	0.02	0.83	**.001**
	False Bay	0.27	0.13	0.14	0.47	**.001**	0.27	0.07	0.19	0.28	**.001**

*Note*: WIC/TNW closer to zero highlights a greater degree of individual specialization, whereas WIC/TNW approaching 1 is characteristic of generalist populations. The significance of the WIC/TNW ratio is denoted by the *p*‐values, generated through a non‐parametric Monte Carlo bootstrap technique. Significant *p*‐values (*p* < 0.05) are highlighted in bold.

## 4 DISCUSSION

Marine predators, particularly when abundant, play an important role in ecosystem structure and function (Young et al., 2015). Consequently, an understanding of their resource‐ and habitat‐use is important from an ecosystem‐based management perspective. Using stable isotope analysis of serially‐sampled whiskers, the present study investigated short‐ and long‐term trends in the trophic ecology of female Cape fur seals breeding at geographically distinct sites in South Africa. We identified geographic gradients and temporal trends in isotopic data across colonies. Furthermore, our results suggest that individual specialization occurs at varying degrees across all breeding sites. While the present study may represent a relatively small sample size in terms of individuals, we obtained a high sample isotopic sample size, which is standard across similar studies (de Lima et al., 2019; Kernaléguen et al., 2012; Kernaléguen, Cherel, et al., 2015).

### 4.1 Inter‐ and intracolony differences

Where marine predators are widely distributed, spatial differences in foraging behavior and diet between separate populations often occur (Baylis et al., 2018; Handley et al., 2017; Staniland et al., 2010). In addition, where breeding colonies differ in population size, foraging behavior, and resource use may further be influenced by density‐dependent competition (Wakefield et al., 2013). Our study found slight but clear differences in the isotopic compositions of female Cape fur seal whiskers from four geographically distinct breeding colonies in South Africa. Similar differences have been documented in other otariid species and are typically ascribed to spatial variation in resource and/or habitat use, or to baseline isotopic shifts in response to oceanographic influences (Baylis et al., 2018; de Lima et al., 2019; Kurle & Worthy, 2001).

Along the South African coast, biogeographic gradients in the isotopic composition of suspended particulate matter and intertidal mussels have previously been highlighted (Hill et al., 2006). Both showed systematic spatial trends, with δ^13^C values, and to a lesser extent δ^15^N values, increasing from the east and south to the west coasts (Hill et al., 2006). Similar isotopic gradients are also apparent at higher trophic levels, with Cape gannets (*Morus capensis*), showing a westward increase in both δ^13^C and δ^15^N values (Jaquemet & McQuaid, 2008), while African black oyster catchers (*Haematopus moquini*) show a westward increase in δ^15^N, but an eastward increase in δ^13^C values (Kohler et al., 2011). Baseline δ^13^C values also show an inshore to offshore decreasing trend across the entire South African coastline (Hill et al., 2006). In the present study, geographic variations in δ^13^C values from female Cape fur seal whiskers are contradictory to the typical westward gradients (Hill et al., 2006; Hill & McQuaid, 2008; Jaquemet & McQuaid, 2008), showing slightly higher values on the east coast than the south east and west coast. This could reflect spatial differences in the foraging habitats utilized by seals from separate breeding sites. Indeed, female Cape fur seals from the easternmost Black Rocks site, and to a lesser extent, False Bay, often forage closer inshore and utilize shallower depth classes compared with females from the westernmost Kleinsee site (Botha et al., 2020). Thus, it is possible that a greater proportion of inshore foraging, particularly for individuals from Black Rocks, could be responsible for the eastward gradient in δ^13^C values.

The slight increase in δ^15^N values of Cape fur seal whiskers from False Bay to Kleinsee are consistent with previous studies (Hill et al., 2006; van der Lingen & Miller, 2014), which suggest a biogeographic increase of baseline δ^15^N values from the south coast to the west coast. This gradient has been attributed to the contrasting influences of the Agulhas and Benguela currents. Interestingly, δ^15^N values from seals at Black Rocks, the eastern‐most colony, did not follow this biogeographic trend and were typically higher than the values for seals from False Bay and Vondeling Island. This could reflect a higher contribution of benthic prey in the diet of Cape fur seals at Black Rocks because benthic prey sources typically exhibit higher δ^15^N values (Moseley et al., 2012). Historically, Cape fur seals from colonies on the south coast were known to consume substantial proportions of benthic prey species such as Cape flounder, *Arnoglossus capensis* and redspotted tongue fish, *Cynoglossus zanzibarensis* (Stewardson, 2001). More recent diet information suggests that seals on the south coast consume a mix of both pelagic and benthic prey species (Connan et al., 2014; Huisamen et al., 2012).

The present study also documented varying degrees of isotopic niche overlap and differences in isotopic niche width among breeding colonies. In general, females from closely located colonies (i.e., False Bay and Vondeling Island) exhibited higher degrees of isotopic niche overlap compared with seals from more distant colonies (e.g., Kleinsee and Black Rocks). This is to be expected given the general baseline differences in both carbon and nitrogen ratios along the southern African coast (Hill et al., 2006). However, trophic niche width differences between study sites were not related to proximity, with the colonies at False Bay and Kleinsee exhibiting substantially larger niche widths than the Vondeling Island and Black Rocks colonies. In addition, trophic niche widths of individuals within colonies were typically more dispersed and showed less overlap at Kleinsee and False Bay, while individuals at Vondeling Island and Black Rocks generally showed less dispersion and substantial overlap. It is possible that the observed inter‐ and intracolony differences in isotopic niche width are influenced by population size. In particular, larger breeding colonies may occupy a broader overall niche area, while individuals from larger breeding colonies may show greater niche segregation in response to higher intraspecific competition (e.g., Svanbäck & Bolnick, 2007). While this is a reasonable explanation for niche width differences between the populations at Kleinsee and Black Rocks, given their differences in population size, it does not explain differences between the populations at Vondeling Island and False Bay which are similar in size.

An alternative explanation is that the geographic patterns observed in the present study are largely influenced by differences in habitat heterogeneity and prey availability. Indeed, the influence of ecological setting on population and individual niche dynamics has been well‐documented across a range of taxa (e.g. Darimont et al., 2009; Heggenes et al., 1999; Kernaléguen, Arnould, et al., 2015; Schriever & Williams, 2013; Simpfendorfer et al., 2001; Yurkowski et al., 2016). The isotopic niche widths of female Cape fur seals in this study were directly proportional to the adjacent shelf and shelf slope area at each colony. Both Kleinsee and False Bay, the colonies with the broadest isotopic niche widths, are located close to broad shelf areas with relatively gently inclining slopes (Figure 1). By contrast, the shelf area around the Vondeling Island and Black Rocks colonies is relatively narrow and the shelf slope relatively steep (Figure 1). Throughout their range, female Cape fur seals are known to forage almost exclusively over the continental shelf and shelf slope (Botha et al., 2020; Skern‐Mauritzen et al., 2009). Therefore, increased ecological opportunity associated with the broader shelf and shelf‐slope areas off the Kleinsee and False Bay colonies may have contributed to observed niche width differences between the four breeding sites.

### 4.2 Periodicity in isotopic signatures

The marine environment is often dynamic, with changes in physical oceanographic conditions known to influence the distribution and availability of prey resources to predators (Dorman et al., 2015; Fiedler & Bernard, 1987). In addition, life cycles of prey species (recruitment, spawning) further impact on their availability at different spatiotemporal scales (Croxall et al., 1985). Marine predators are expected to alter their foraging in response to such heterogeneity and consequently, temporal differences in foraging effort (Angel et al., 2015; Harding et al., 2007), behavior (Botha & Pistorius, 2018; Foo et al., 2019) and diet (Chambellant et al., 2013; Reisinger et al., 2018) have been well documented.

In the present study, whisker growth rates of female Cape fur seals were estimated to be between 0.06 and 0.1 mm.day^−1^, such that each 3 mm segment is estimated to represent a period of 38.9 d on average. This is within the range previously determined for female Antarctic, subantarctic, and Australian fur seals (Cherel et al., 2009; Kernaléguen et al., 2012, 2016). The periodic oscillations documented in δ^13^C and δ^15^N values along the whisker length indicate that, as with other fur seal species, Cape fur seal females from South African breeding colonies exhibit similar temporal variability in isotopic composition. This may reflect inter‐ and intra‐annual trends in the movement and diet of individuals in relation to fluctuations in prey availability. Alternatively, this could also reflect broadscale shifts in the distributions and abundance of several forage species including anchovy and sardine that have previously been documented for the Benguela ecosystem (Coetzee et al., 2008; Roy et al., 2007). These have had far reaching implications for foraging behavior and diet of several seabird species throughout the region, particularly on South Africa's west coast (Crawford et al., 2011, 2016; Grémillet et al., 2008). In comparison, seabirds breeding at colonies on the south and south‐east coasts appear to have benefitted from the eastward movement of pelagic prey species, particularly anchovy (Crawford et al., 2009; Green et al., 2015). Interannual trends in isotopic compositions of Cape fur seal whiskers in the present study may also be linked to shifts in the distribution and abundances of pelagic forage species. Further investigation, possibly drawing on scat analyses, eDNA and/or stable isotope mixing models, to infer diet composition, is needed to clarify this issue (e.g., Bjorkland et al., 2015; Handley et al., 2017).

### 4.3 Individual specialization

With increasing evidence of individual foraging specialization within wild animal populations (Bearhop et al., 2006; Matich et al., 2011; Robertson et al., 2014; Schriever & Williams, 2013), it is likely that many populations often comprise generalist and specialist individuals (Bolnick et al., 2003). Although Cape fur seals have previously been described as generalist foragers (e.g., David, 1987; Huisamen et al., 2012), our results indicate that specialist individuals may be present in certain populations, and may consistently use only a subset of a population's niche width. Specifically, Roughgarden's indices revealed that, across colonies, the degree of specialization was always higher for trophic (δ^15^N) than for spatial (δ^13^C) dimensions, which suggests that females may specialize on certain prey types across a variety of habitats. This is consistent with Roughgarden's indices of female Antarctic and South American fur seals (de Lima et al., 2019; Jones et al., 2020) but in contrast with previous findings in female Australian fur seals, which typically show a higher degree of specialization across δ^13^C values (Kernaléguen, Cherel, et al., 2015).

For female Cape fur seals in the present study, observed individual specialization was, however, not consistent across colonies, with Roughgarden's indices indicating higher levels of specialization in δ^15^N values at Kleinsee and False Bay compared with Vondeling Island. Although the drivers of individual specialization are often difficult to ascertain, the influence of population size and ecological opportunity have become central to understanding individual level differences in resource and habitat use (Araújo et al., 2011). It is possible that increased levels of intraspecific competition at larger colonies may lead to increased individual level differences in habitat and resource selection as a means of resource partitioning. As for the observed niche width differences, this may explain the higher degree of specialization recorded at the larger Kleinsee colony, but fails to explain the differences between False Bay and Vondeling Island which are similar in size. It could be that False Bay animals experience competition from other, similar‐sized colonies in its general vicinity (e.g., Geyser Rock), but this explanation seems unsatisfactory given that Vondeling Island is also located close to other dense Cape fur seal colonies. This brings into question the potential impact of ecological setting, particularly the influence of greater resource and/or habitat diversity. Several studies have documented clear links between individual specialization and habitat/resource heterogeneity (Heggenes et al., 1999; Newsome et al., 2015; Yurkowski et al., 2016).

Regardless of the underlying drivers, individual specialization has become an important consideration in ecological studies because it may have considerable fitness implications (Authier et al., 2012; Cucherousset et al., 2011; Franco‐Trecu et al., 2014; Patrick & Weimerskirch, 2014). From a planning and management perspective, identifying specialization within wild populations is of further importance, especially when predicting responses to future environmental and anthropogenic changes, as well as managing human–wildlife interactions (Bearhop et al., 2004; Votier et al., 2010). This information aids in identifying specific high risk populations and individuals and allows for better predictions surrounding the future response of marine predators to environmental change (e.g., Lawton et al., 2012). Given that Cape fur seals comprise a substantial proportion of South Africa's marine predator biomass, and with concerns around fisheries interaction and competition (David, 1987), future work should include a focus on individuals within populations and broadscale assessments.

## 5 CONCLUSION

The present study provides important baseline data on the isotopic ecology of female Cape fur seals breeding in South Africa. Our results indicate that both resource and habitat use show some degree of geographic and temporal variation. Furthermore, our results suggest that although largely a generalist species, some populations contain individual specialists that use only a subset of the total population niche. This is an advancement in our understanding of the foraging ecology of this functionally important marine top predator. Future studies on Cape fur seal diet would benefit from combining traditional methods, such as scat analysis, with indirect measures including stable isotope, fatty acid and DNA analysis (e.g., Jeanniard‐du‐Dot et al., 2017). This information, particularly when collected over broad spatiotemporal scales, will assist with further interpretation of the results of the current study. Finally, future studies should include adult males and juveniles into their assessments, to further enhance our understanding of this functionally important marine predator.

## AUTHOR CONTRIBUTIONS

**Jonathan A. Botha:** Conceptualization (lead); formal analysis (lead); funding acquisition (equal); investigation (lead); methodology (lead); visualization (lead); writing – original draft (lead); writing – review and editing (equal). **Clive N. Trueman:** Conceptualization (equal); funding acquisition (equal); investigation (equal); supervision (equal); writing – review and editing (equal). **Stephen P. Kirkman:** Investigation (equal); methodology (equal); supervision (equal); writing – review and editing (equal). **John P. Y. Arnould:** Conceptualization (equal); funding acquisition (equal); investigation (equal); methodology (equal); writing – review and editing (equal). **Amanda T. Lombard:** Funding acquisition (equal); supervision (equal); writing – review and editing (equal). **Maëlle Connan:** Methodology (equal); writing – review and editing (equal). **G. J. Greg Hofmeyr:** Data curation (equal); methodology (equal); writing – review and editing (equal). **S. Mduduzi Seakamela:** Data curation (equal); writing – review and editing (equal). **Pierre A. Pistorius:** Conceptualization (equal); funding acquisition (equal); supervision (equal); writing – review and editing (equal).

## CONFLICT OF INTEREST STATEMENT

The authors declare that there are no competing interests

## Supporting information


**Figure A1**Figure A2

## Data Availability

The data that support the findings of this study are made available as electronic supplementary material.
