# Supplementary material for: Geographical, temporal, and individual‐based differences in the trophic ecology of female Cape fur seals
Source: Ecol Evol. 2023 Feb 8;13(2):e9790. doi: 10.1002/ece3.9790 (PMC9909003; doi:10.1002/ece3.9790)
Supplement: Supplementary file 1 — Figure A1Figure A2 [file ECE3-13-e9790-s001.docx]

**Supplementary Appendix**


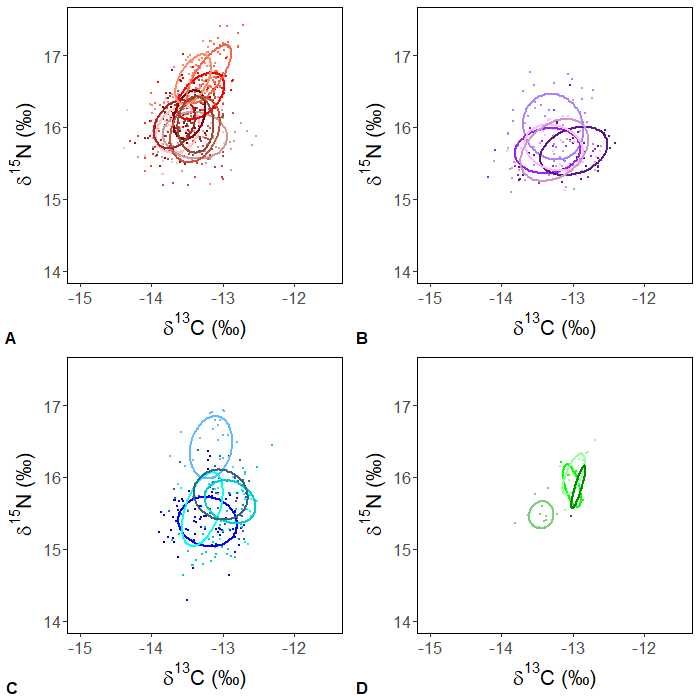


Fig A1 Standard Ellipse Areas corrected for small sample size (SEAc) calculated for individual female Cape fur seals from Kleinsee (A), Vondeling Island (B), False Bay (C) and Black Rocks (D) based on all available whisker fragments. Each individual is presented as a unique colour and points present the δ^13^C and δ^15^N values for each 3 mm whisker fragment.

| Kleinsee Seal 1 | |
| --- | --- |
| δ^13^C | |
| 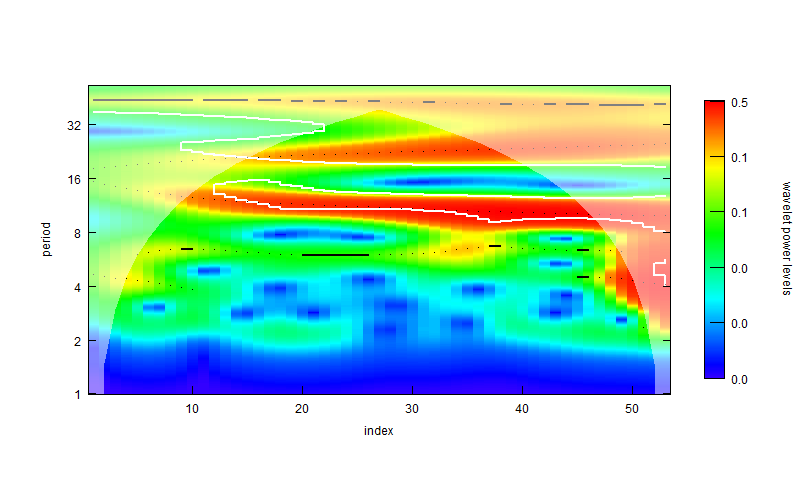 | 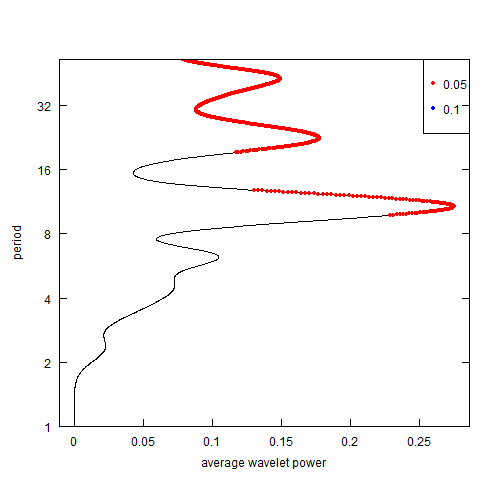 |
| δ^15^N | |
| 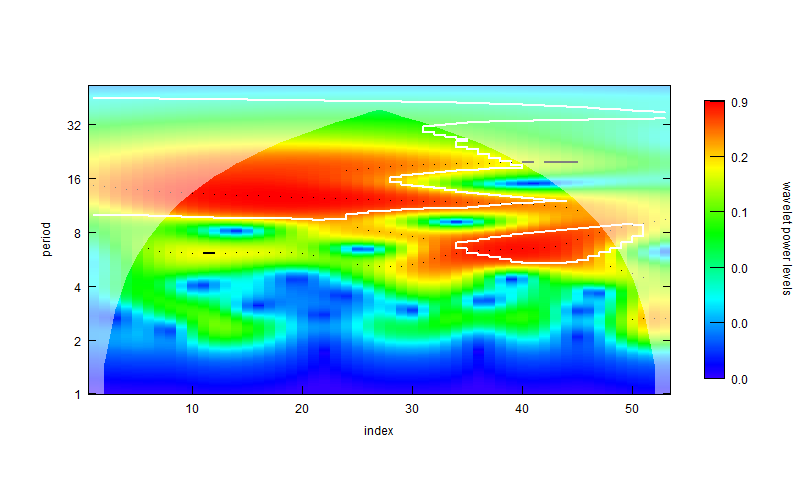 | 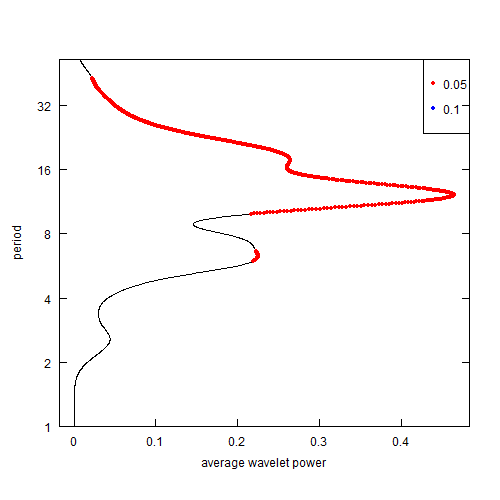 |

| Kleinsee Seal 2 | | | |
| --- | --- | --- | --- |
| δ^13^C | | δ^15^N | |
| 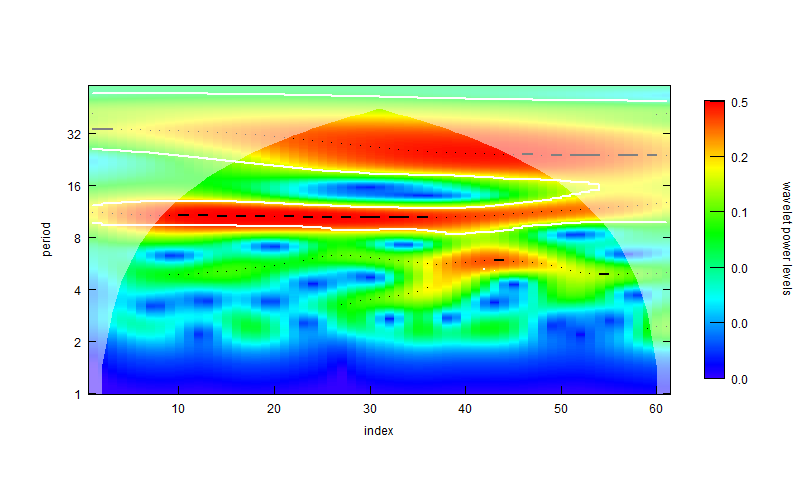 | 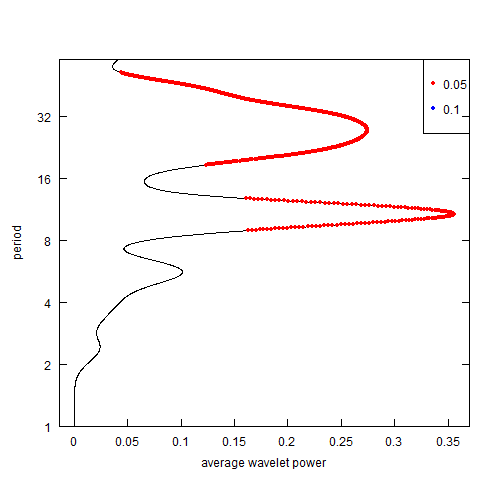 | 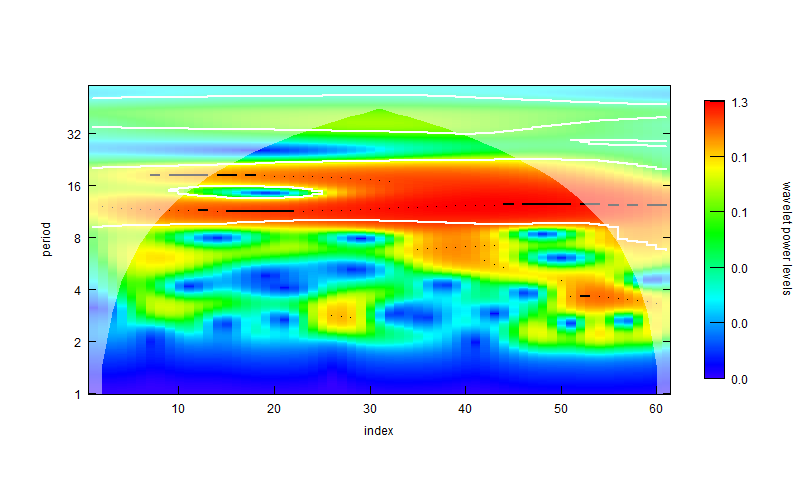 | 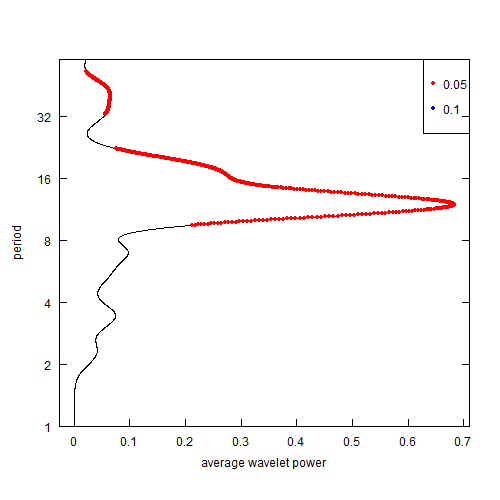 |
| Kleinsee Seal 3 | | | |
| δ^13^C | | δ^15^N | |
| 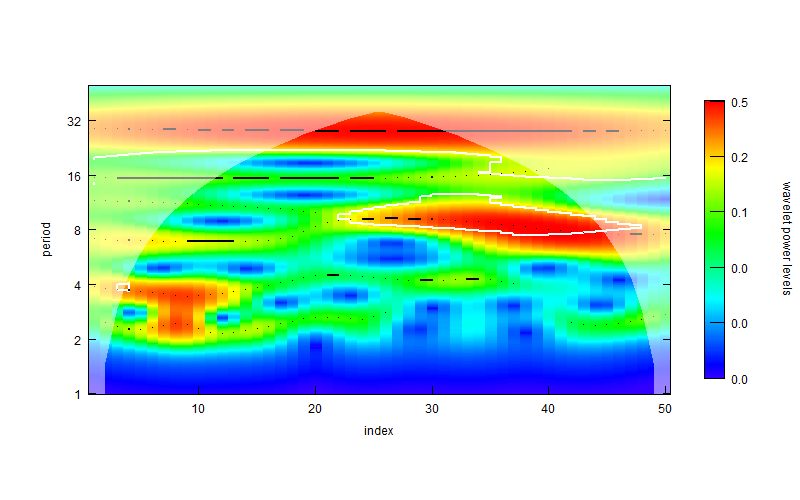 | 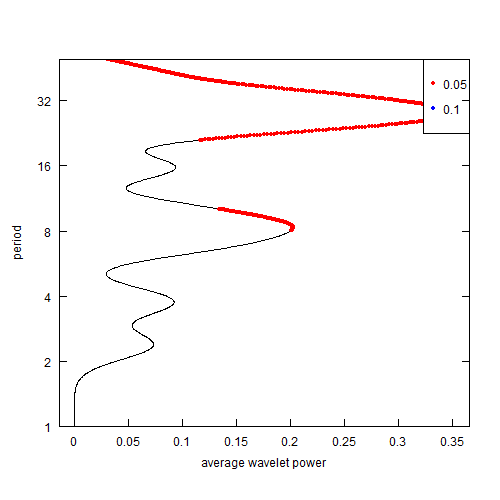 | 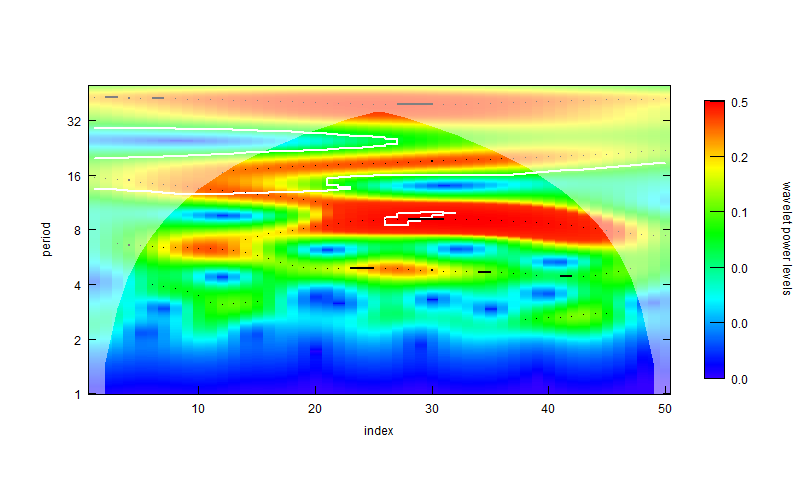 | 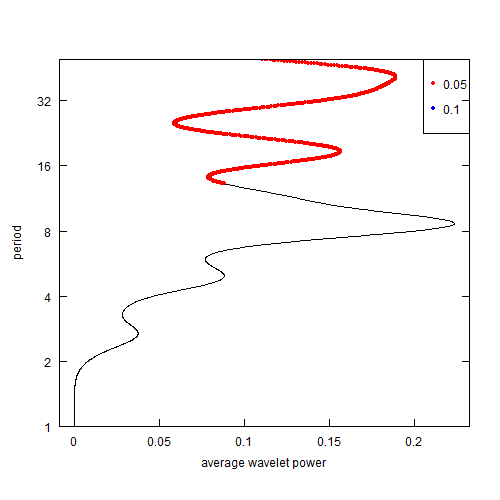 |
| Kleinsee Seal 4 | | | |
| δ^13^C | | δ^15^N | |
| 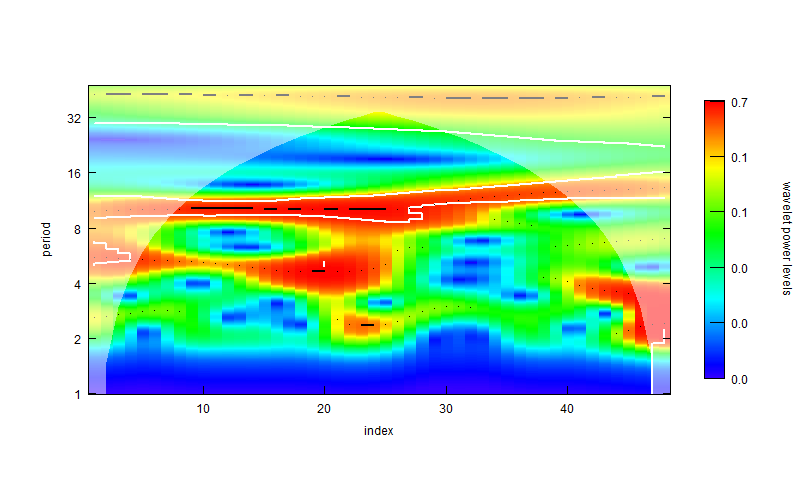 | 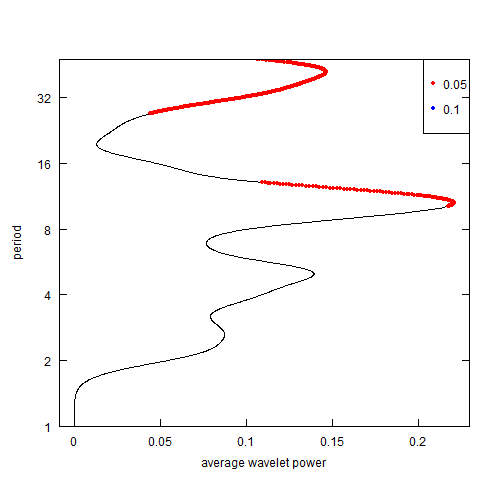 | 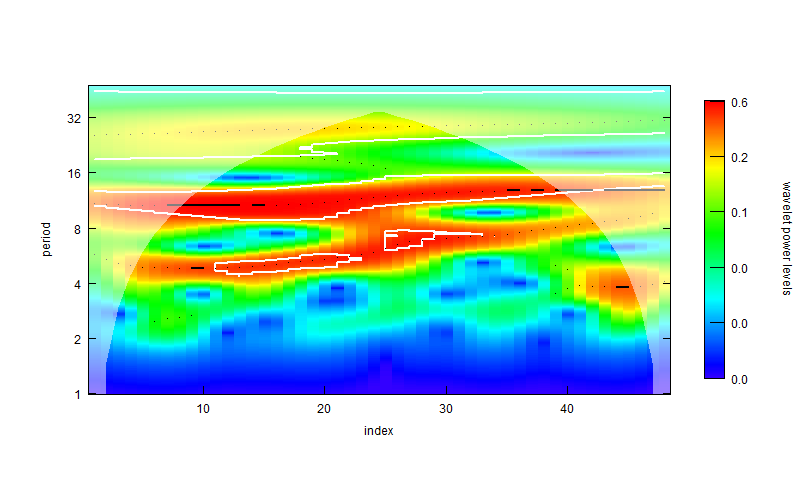 | 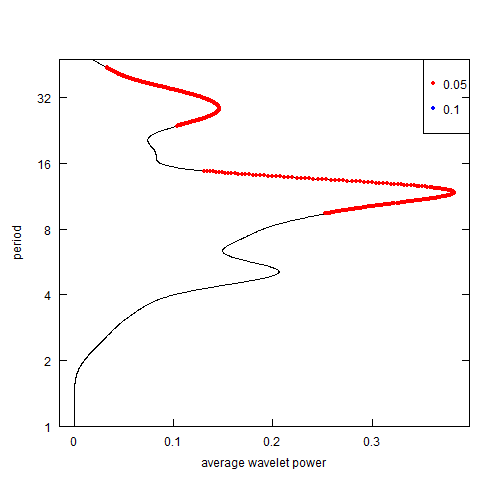 |
|  | | | |
|  | | | |
| Kleinsee Seal 5 | | | |
| δ^13^C | | δ^15^N | |
| 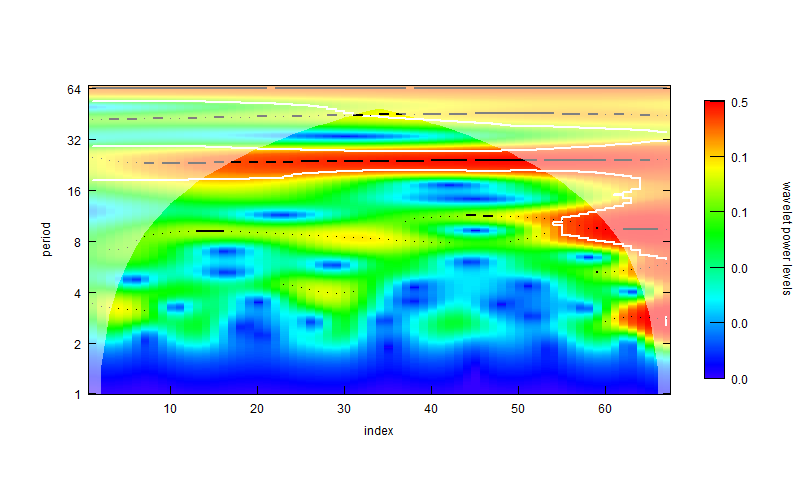 | 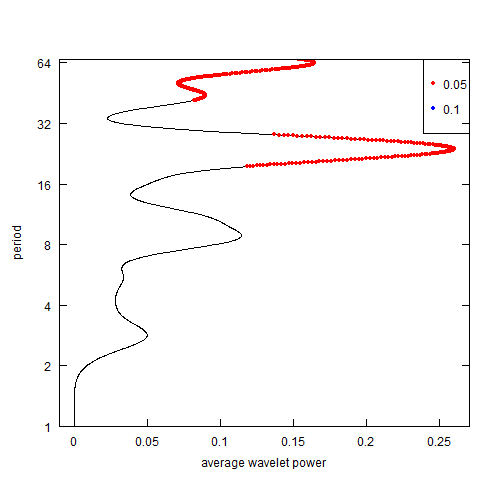 | 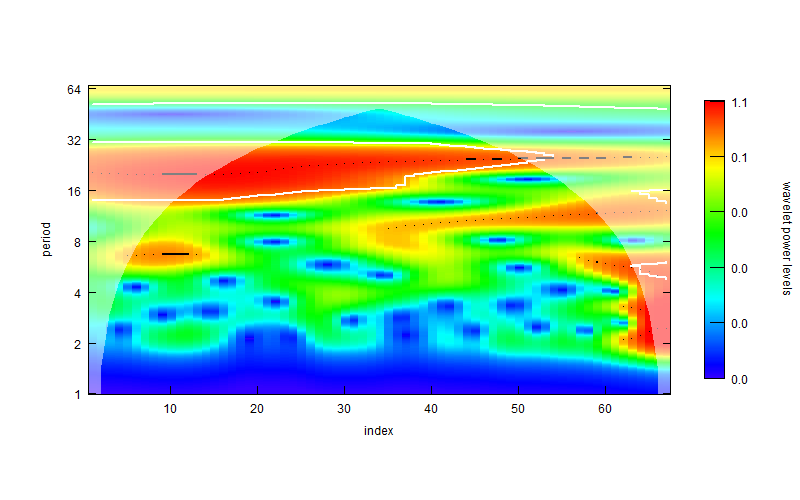 | 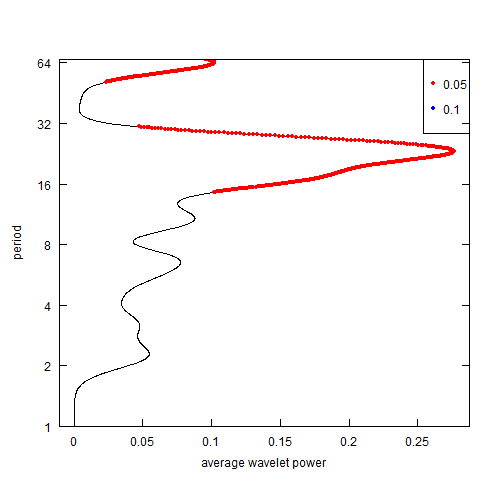 |
|  | | | |
| Kleinsee Seal 6 | | | |
| δ^13^C | | δ^15^N | |
| 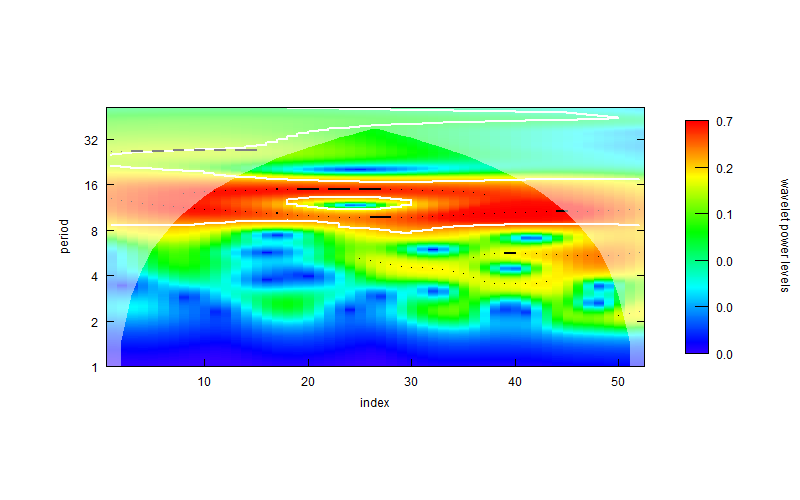 | 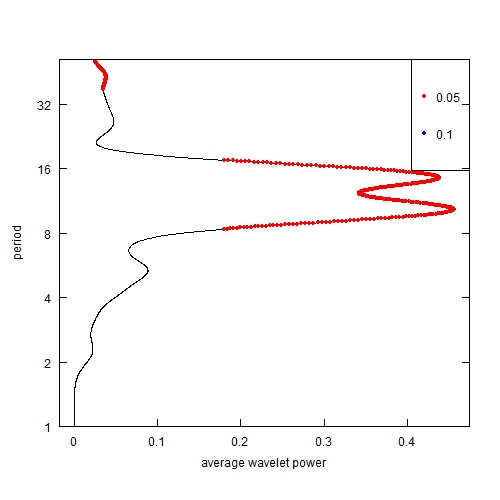 | 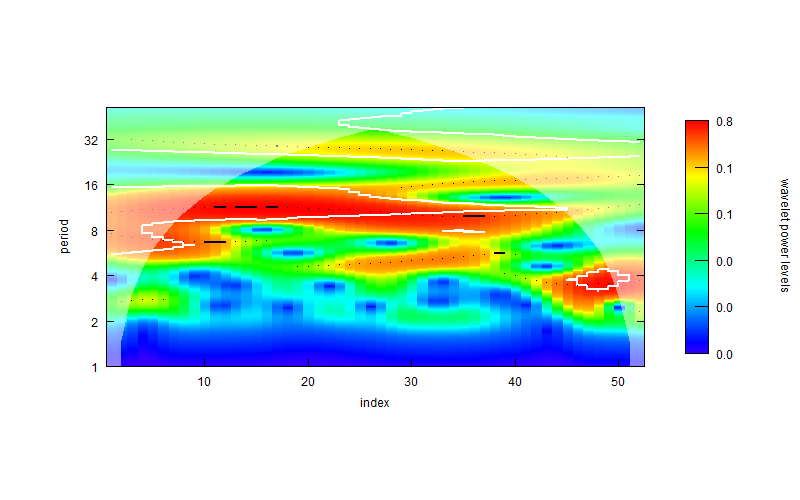 | 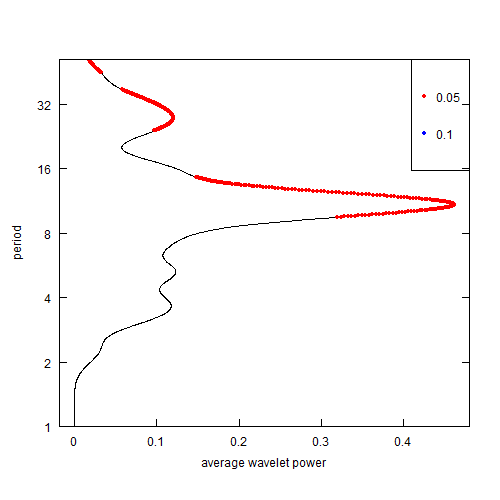 |
| Kleinsee Seal 7 | | | |
| δ^13^C | | δ^15^N | |
| 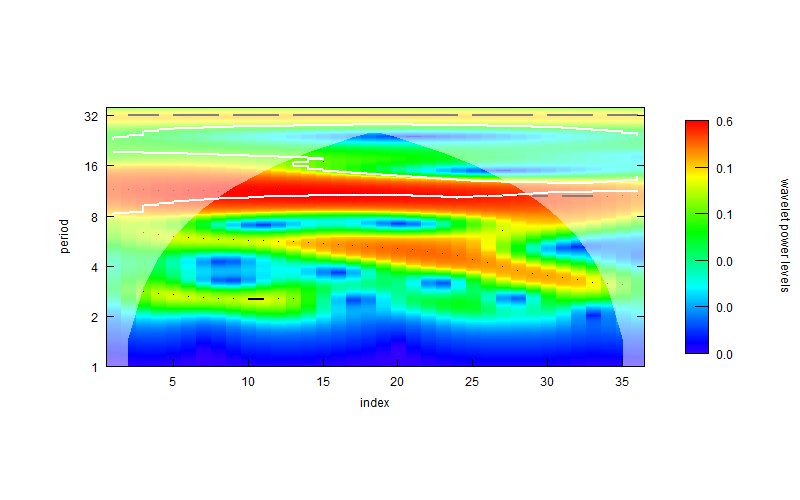 | 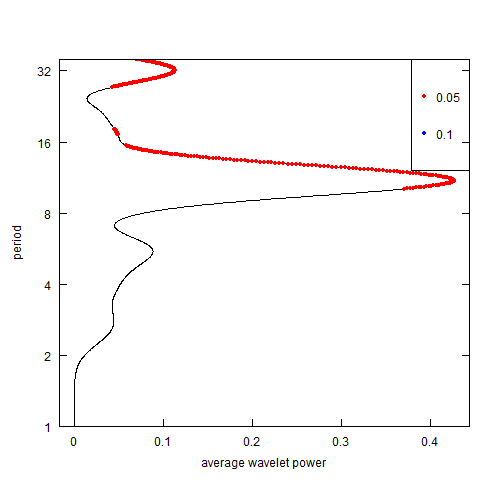 | 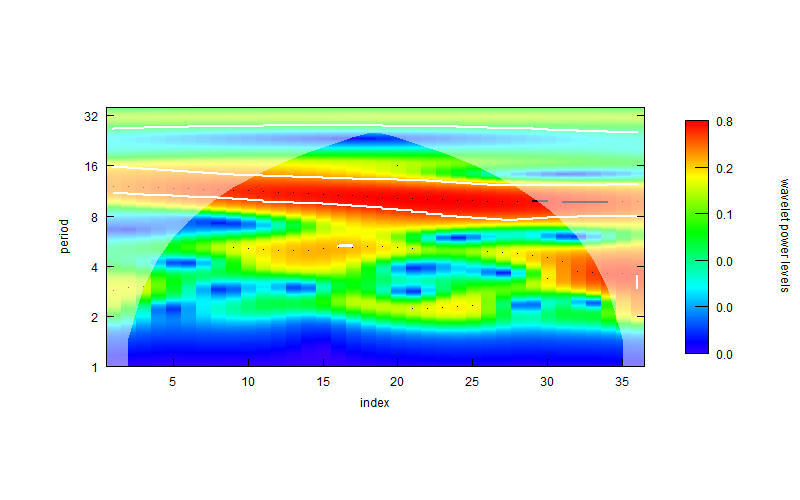 | 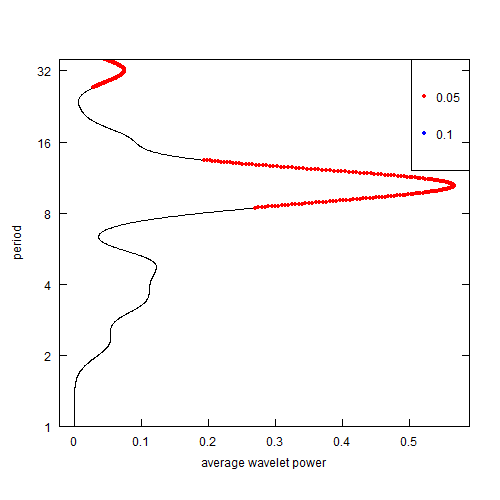 |
|  | | | |
|  | | | |
| Kleinsee Seal 8 | | | |
| δ^13^C | | δ^15^N | |
| 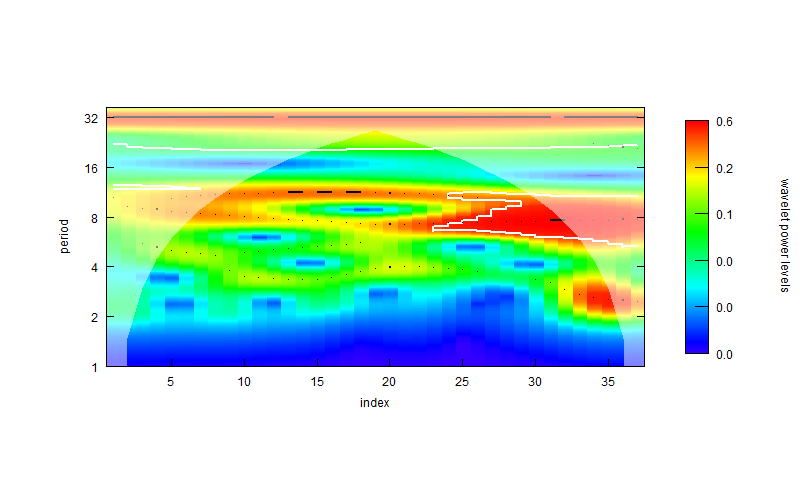 | 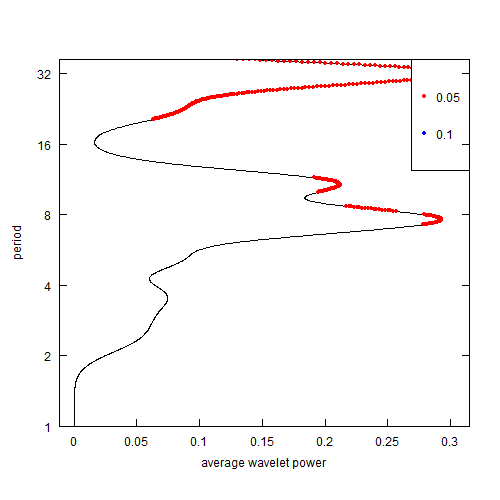 | 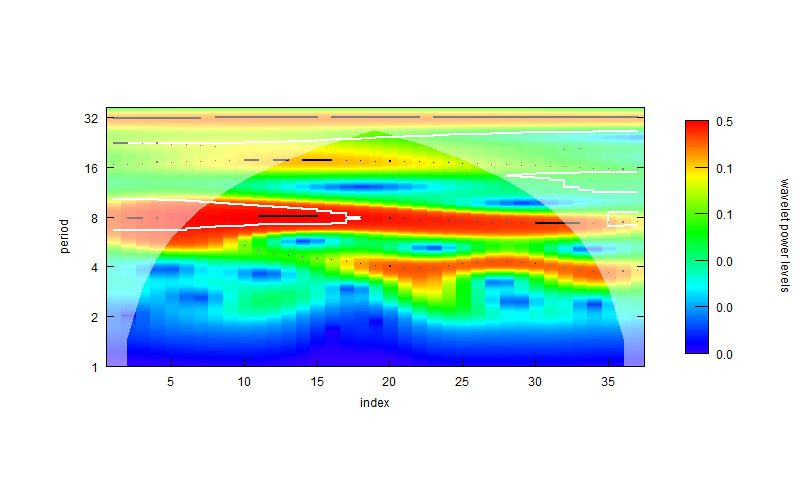 | 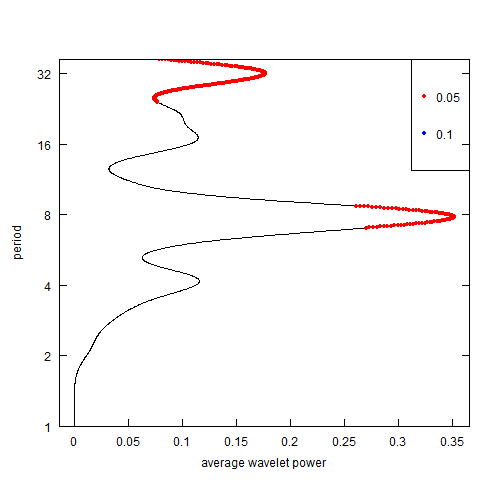 |
| Kleinsee Seal 9 | | | |
| δ^13^C | | δ^15^N | |
| 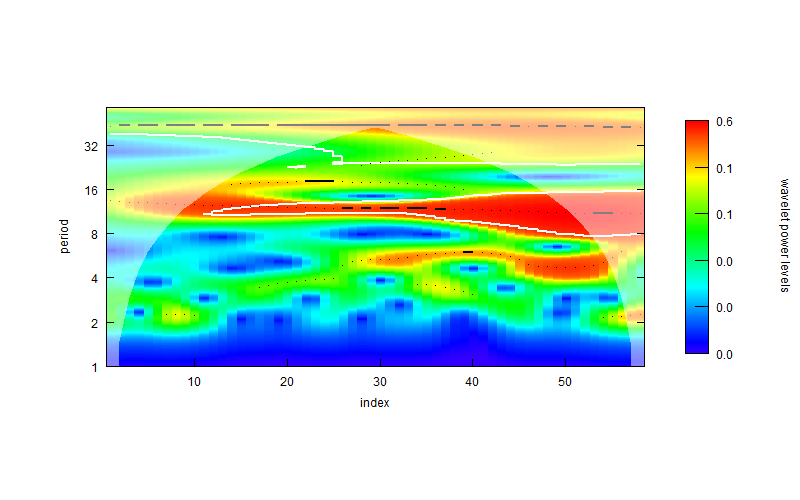 | 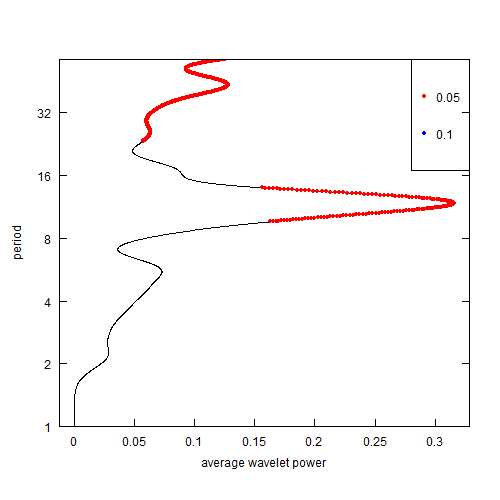 | 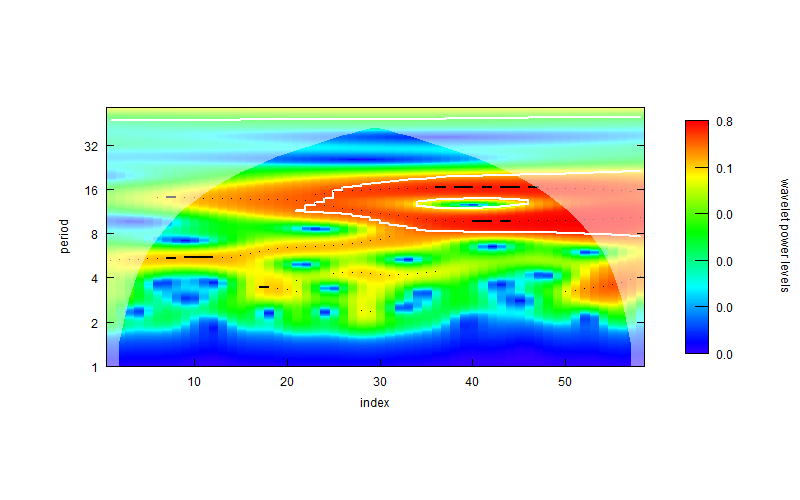 | 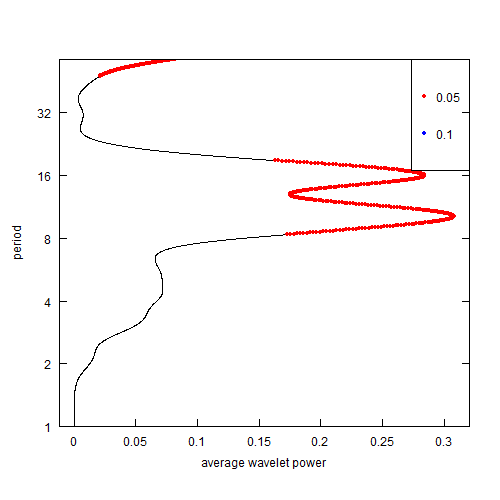 |
| Kleinsee Seal 10 | | | |
| δ^13^C | | δ^15^N | |
| 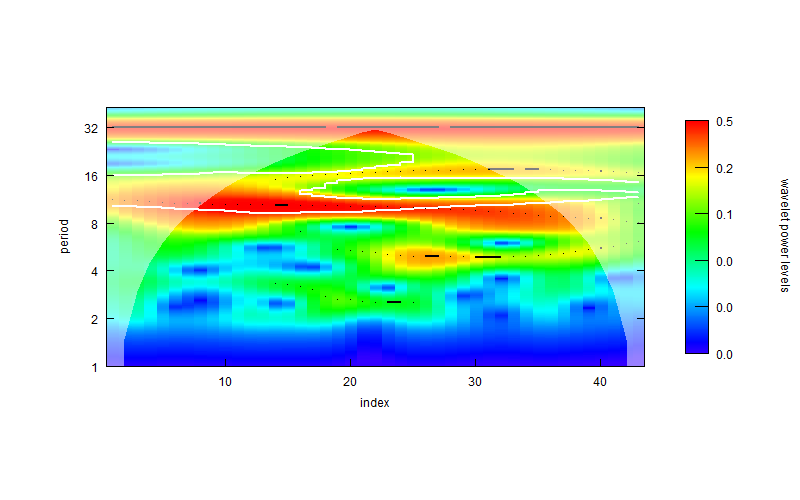 | 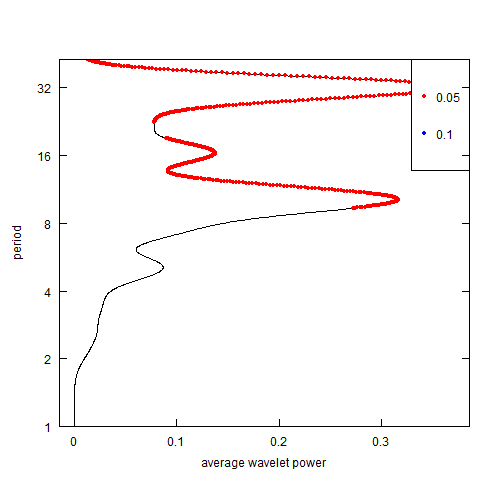 | 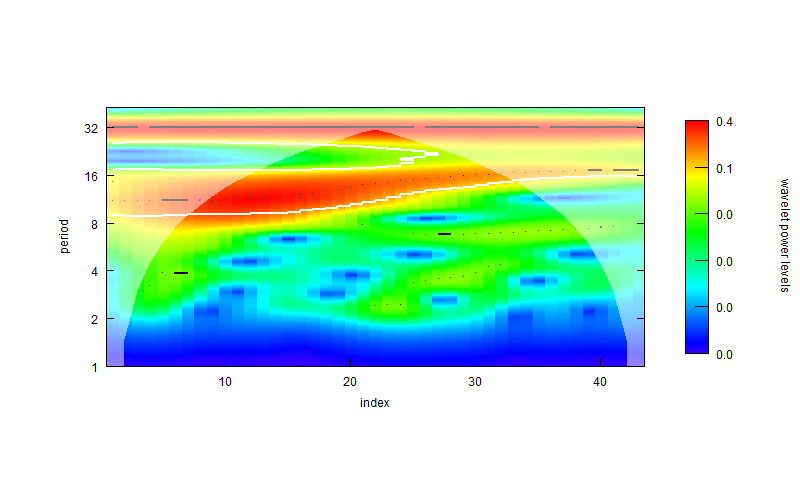 | 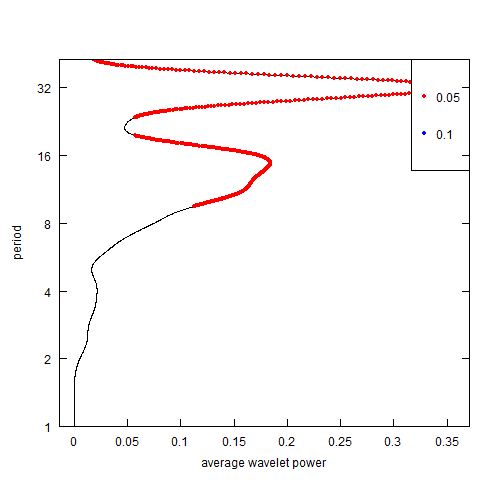 |
|  | | | |
|  | | | |
|  | | | |
| Vondeling Island Seal 11 | | | |
| δ^13^C | | δ^15^N | |
| 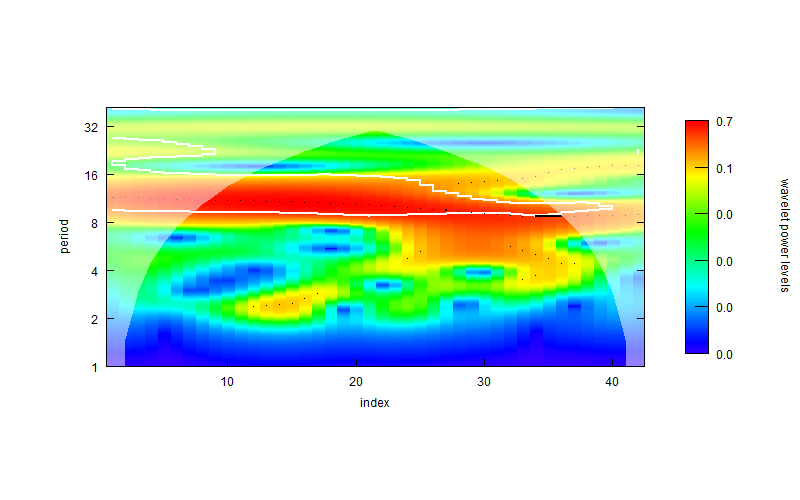 | 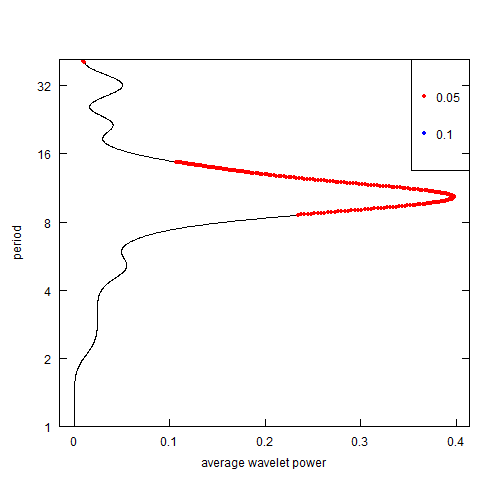 | 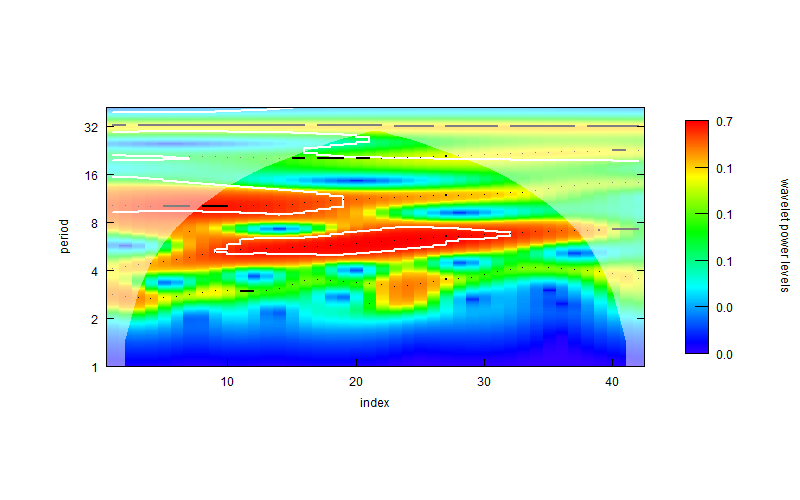 | 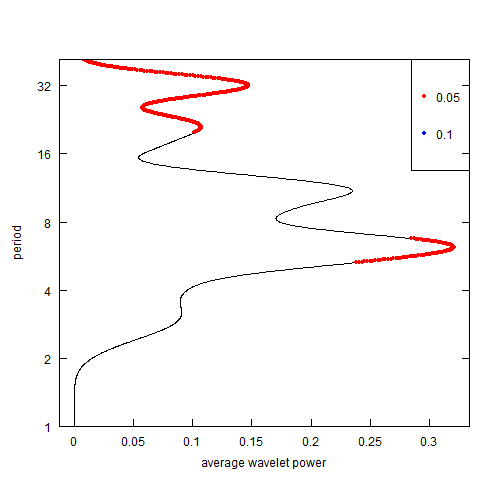 |
| Vondeling Island Seal 12 | | | |
| δ^13^C | | δ^15^N | |
| 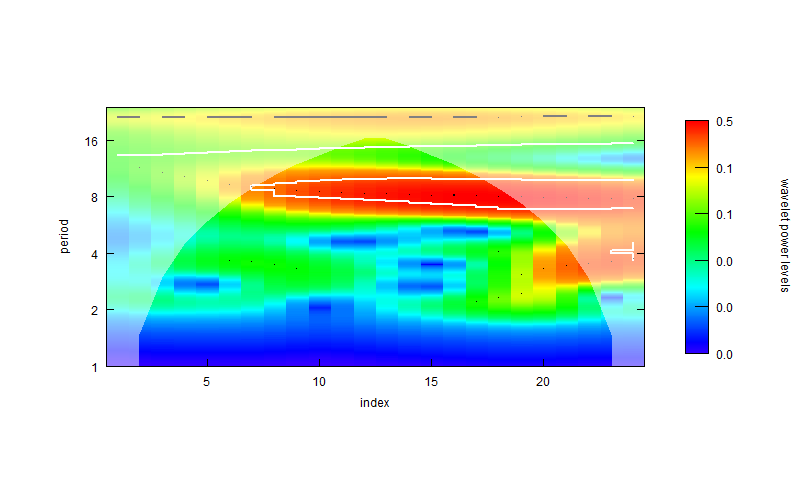 | 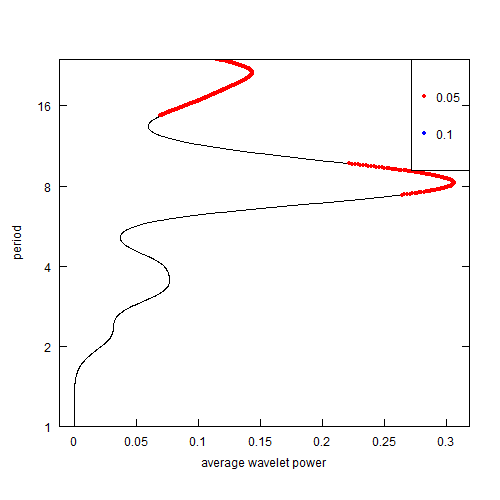 | 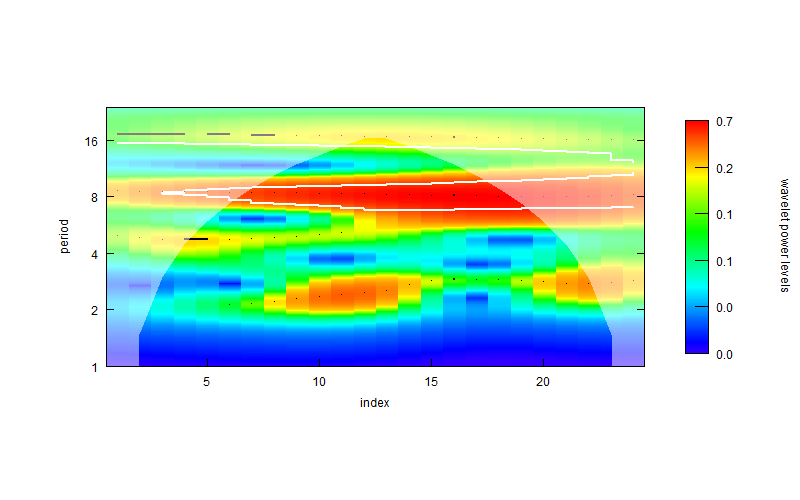 | 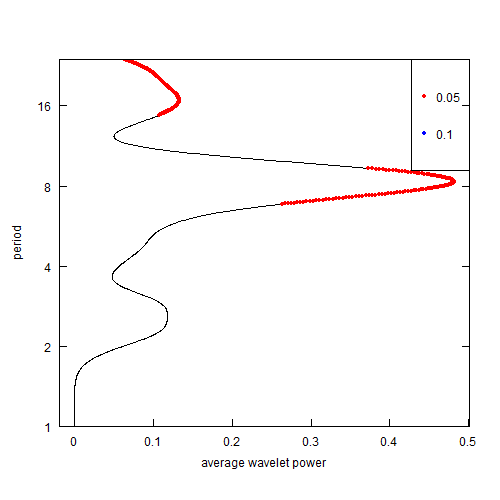 |
| Vondeling Island Seal 13 | | | |
| δ^13^C | | δ^15^N | |
| 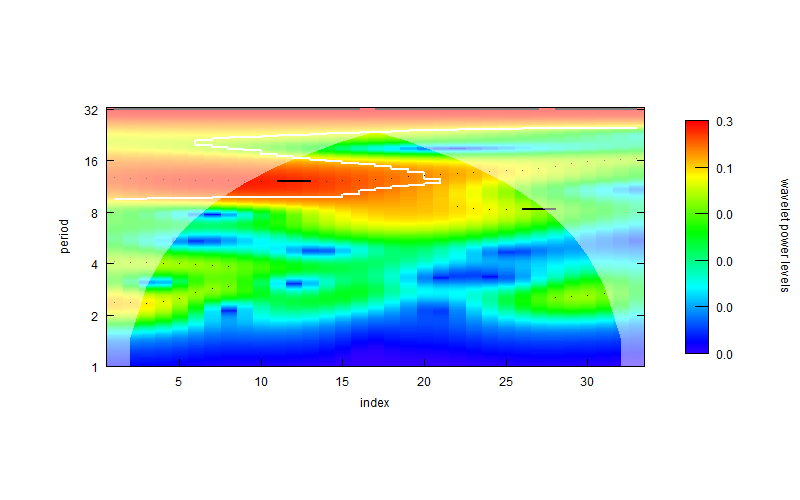 | 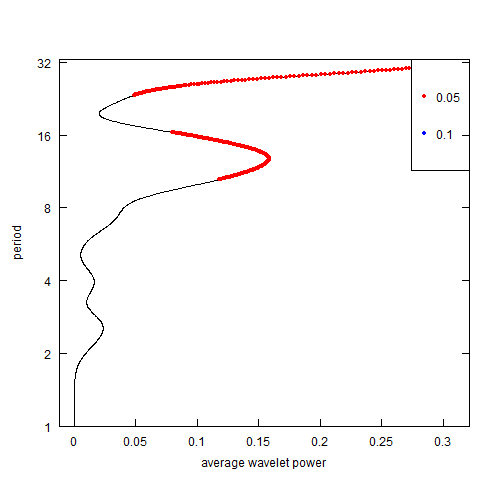 | 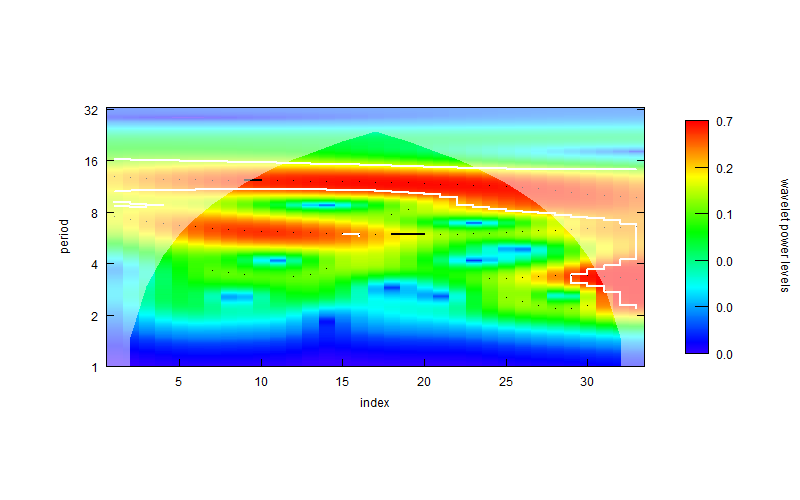 | 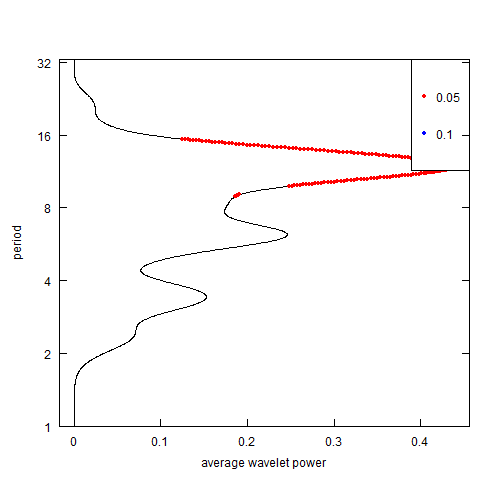 |
|  | | | |
|  | | | |
|  | | | |
| Vondeling Island Seal 14 | | | |
| δ^13^C | | δ^15^N | |
| 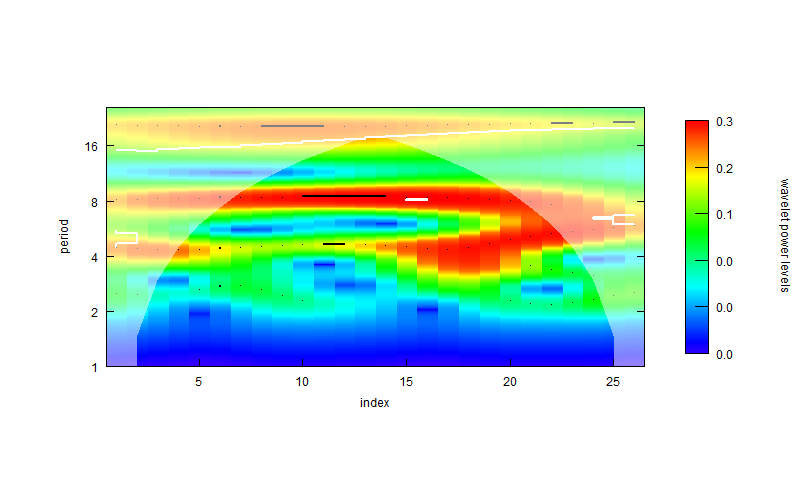 | 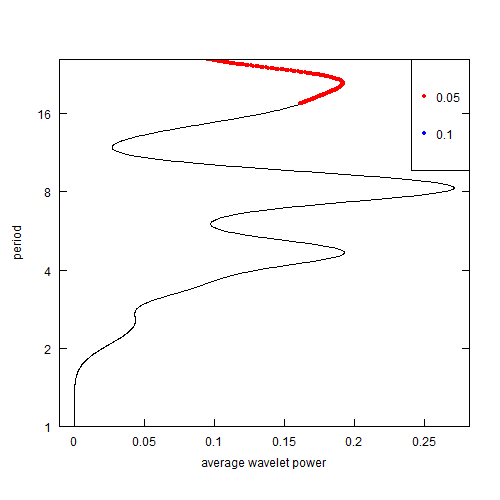 | 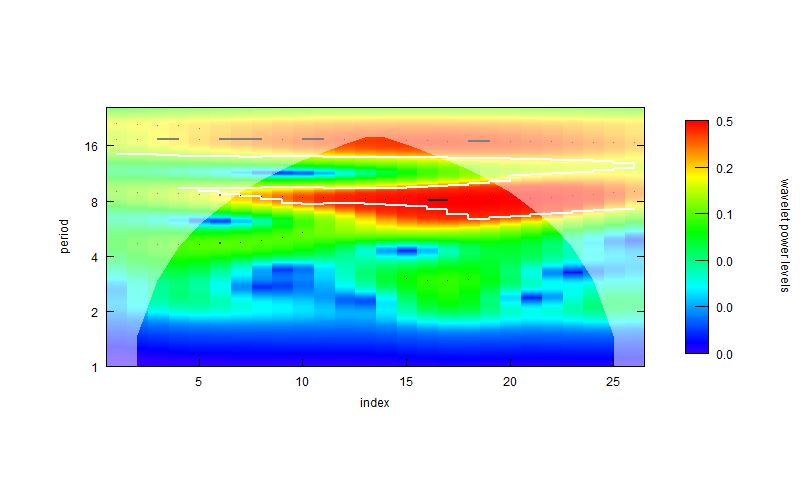 | 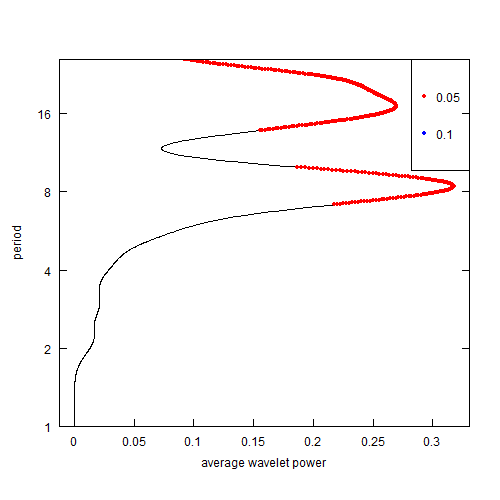 |
| Vondeling Island Seal 15 | | | |
| δ^13^C | | δ^15^N | |
| 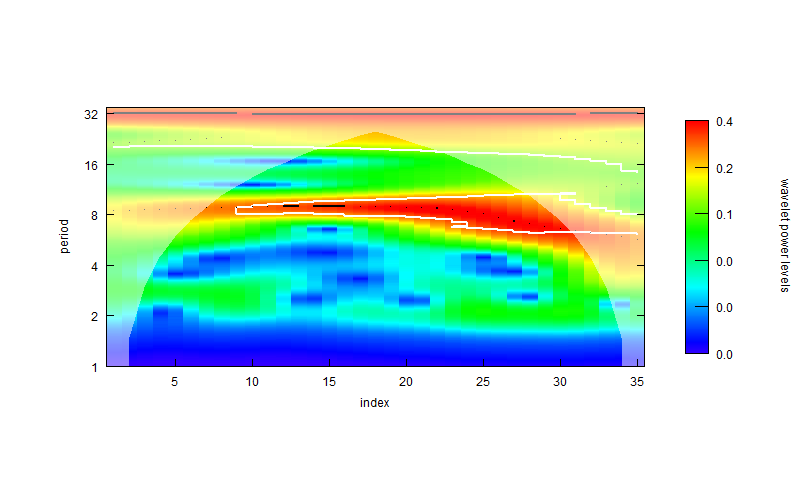 | 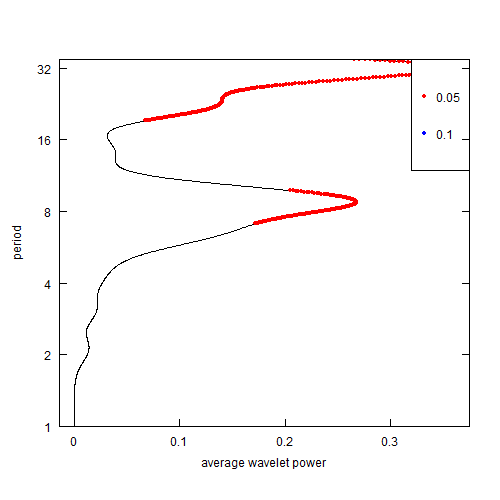 | 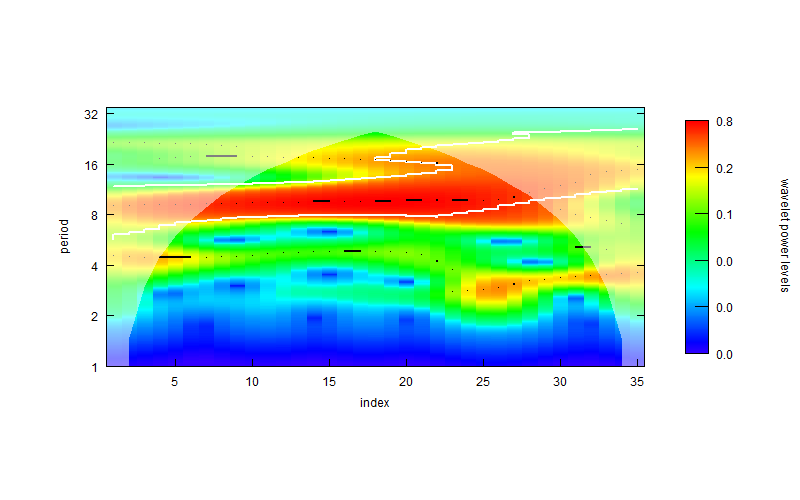 | 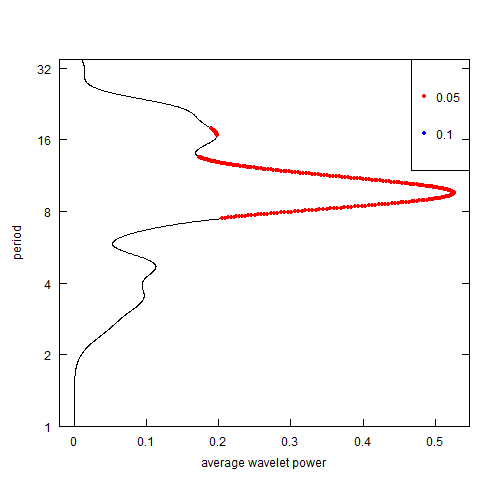 |
| False Bay Seal 16 | | | |
| δ^13^C | | δ^15^N | |
| 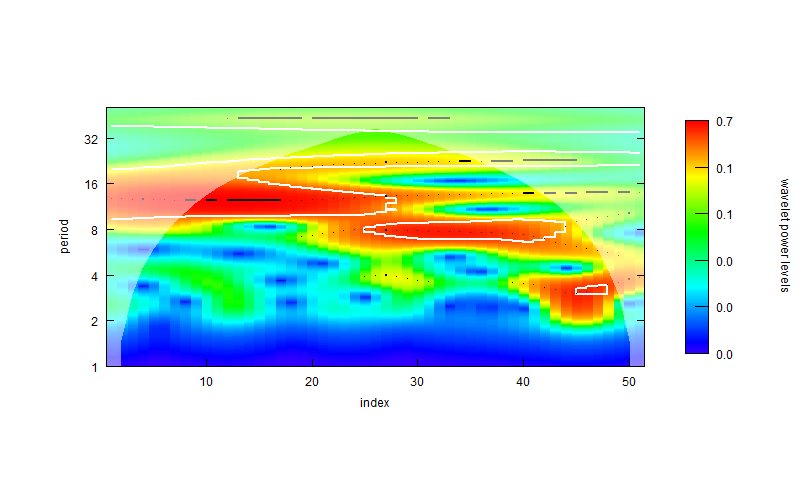 | 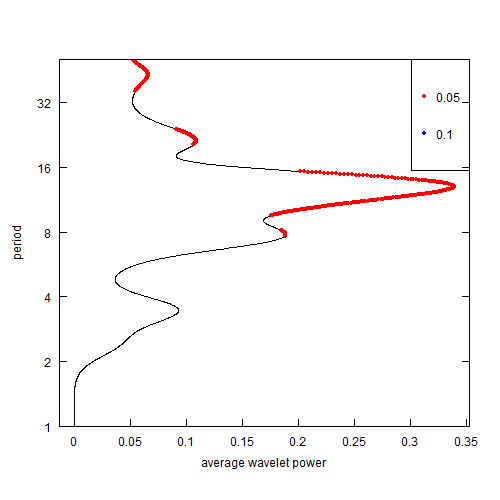 | 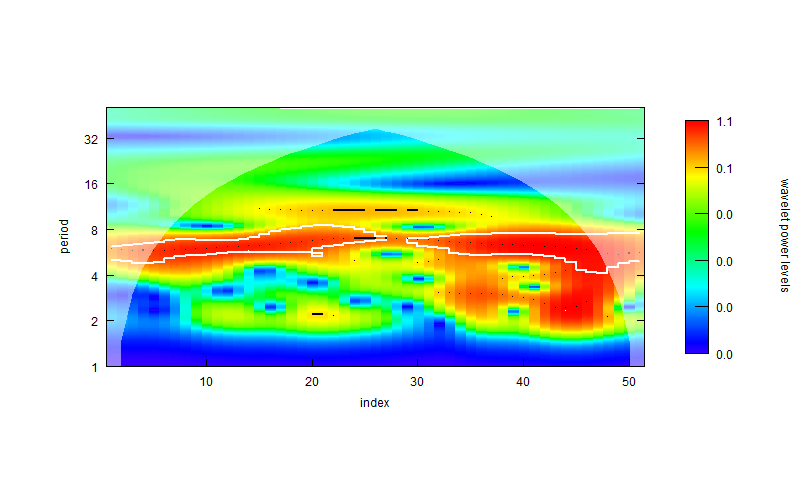 | 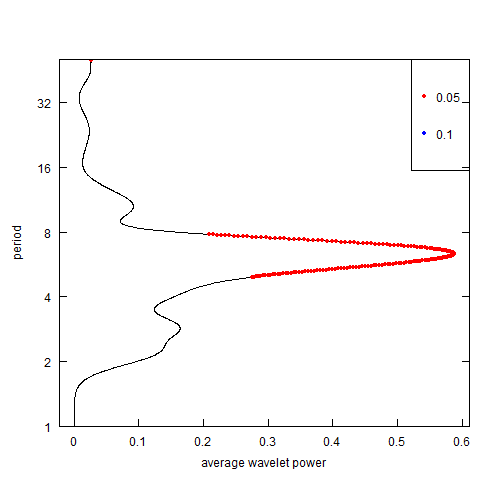 |
|  | | | |
|  | | | |
|  | | | |
| False Bay Seal 17 | | | |
| δ^13^C | | δ^15^N | |
| 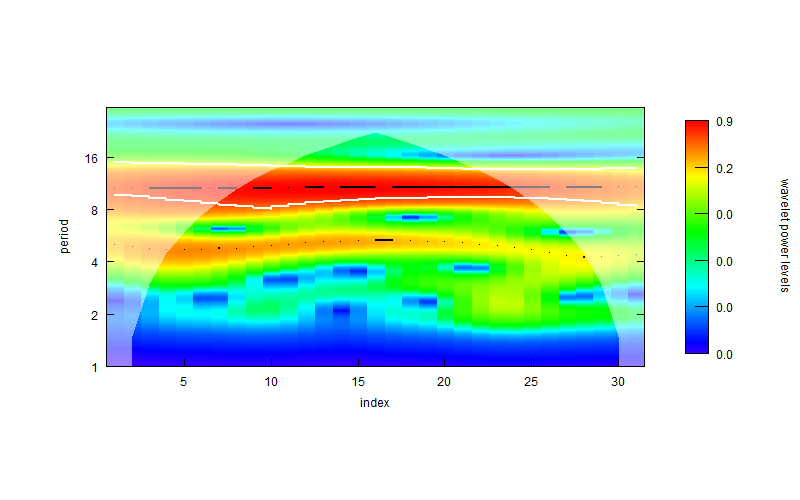 | 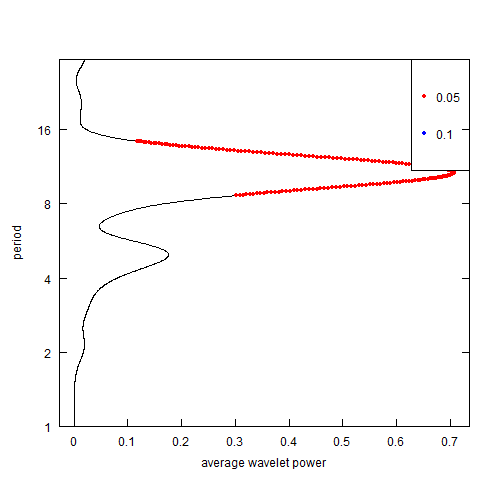 | 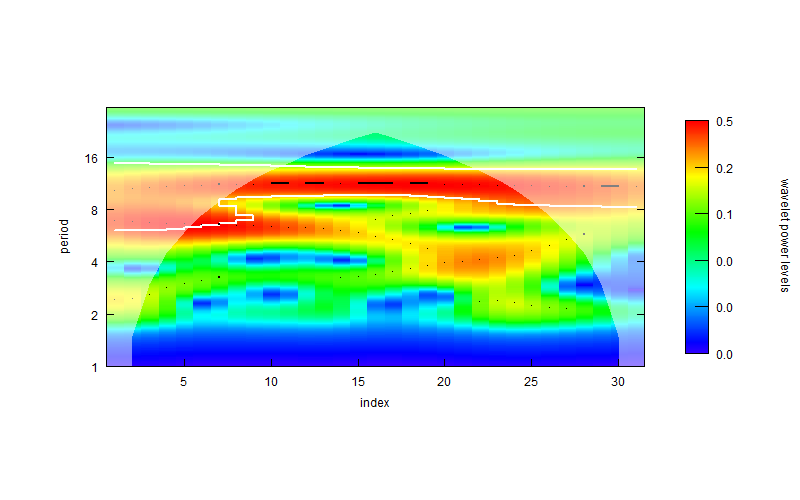 | 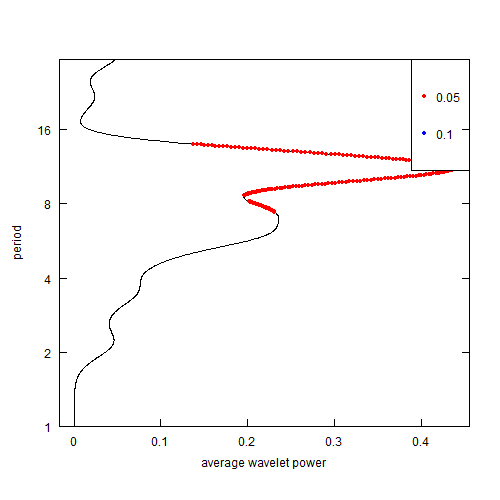 |
| False Bay Seal 18 | | | |
| δ^13^C | | δ^15^N | |
| 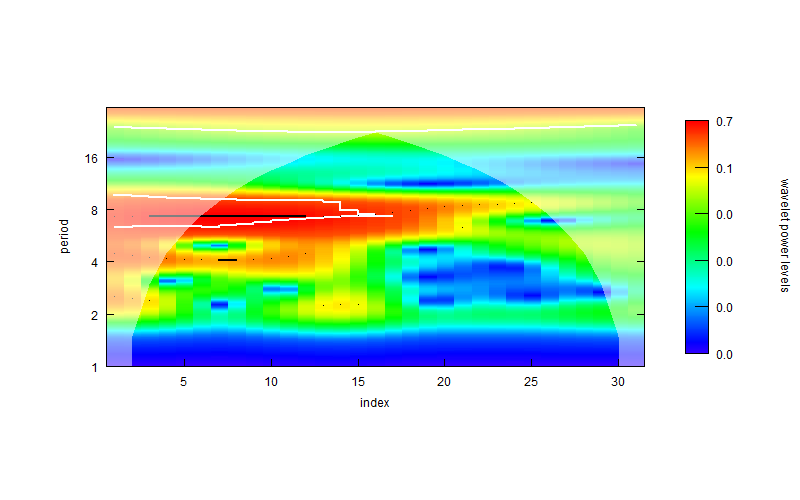 | 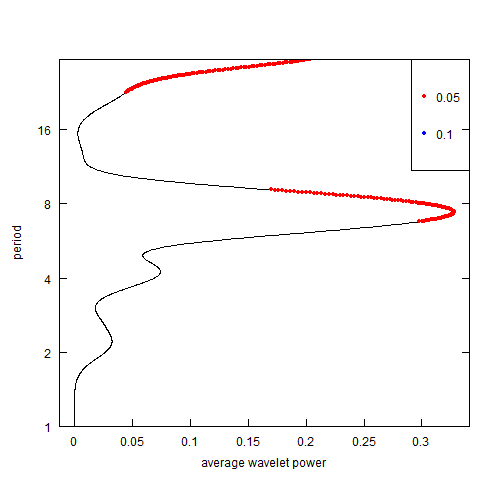 | 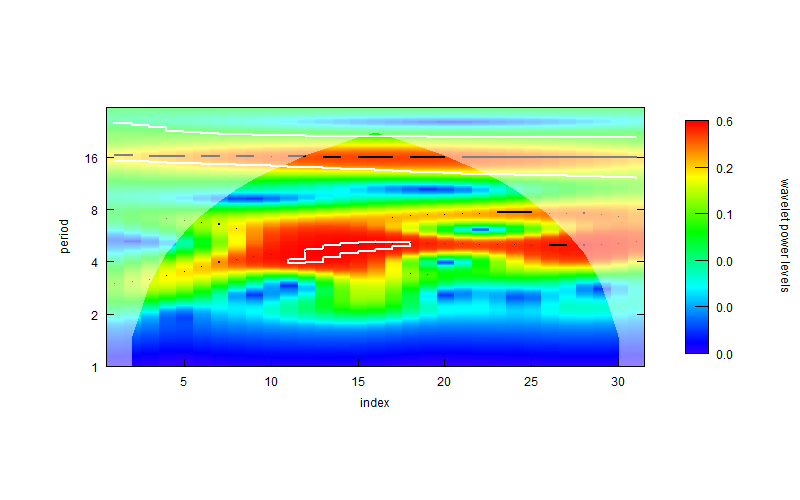 | 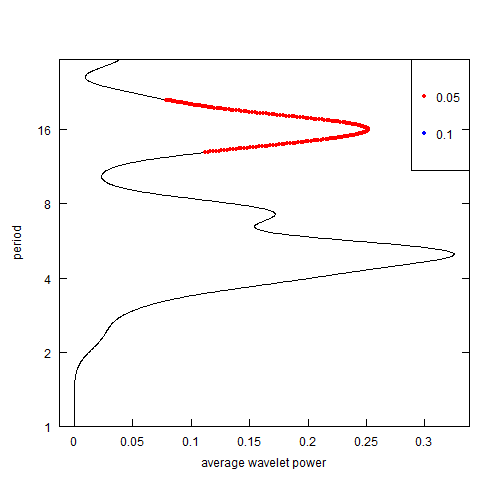 |
| False Bay Seal 19 | | | |
| δ^13^C | | δ^15^N | |
| 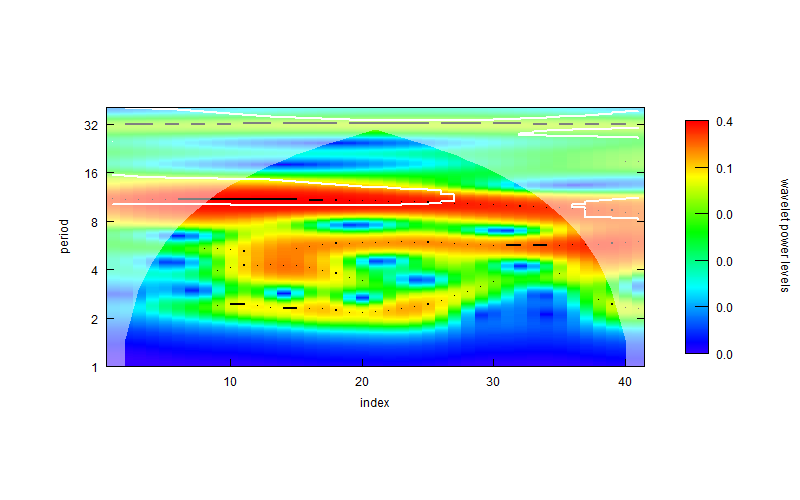 | 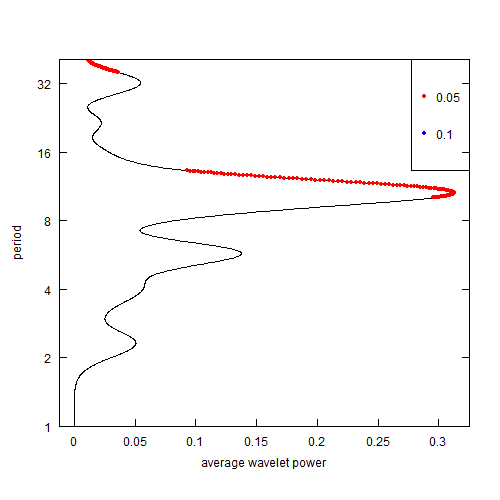 | 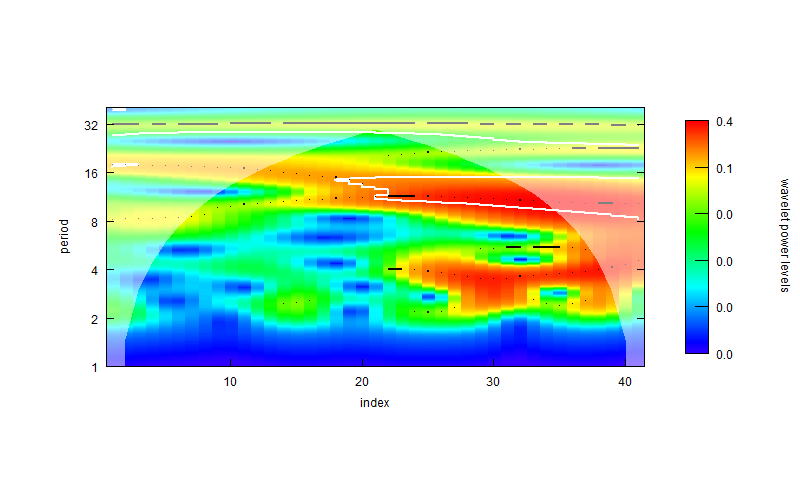 | 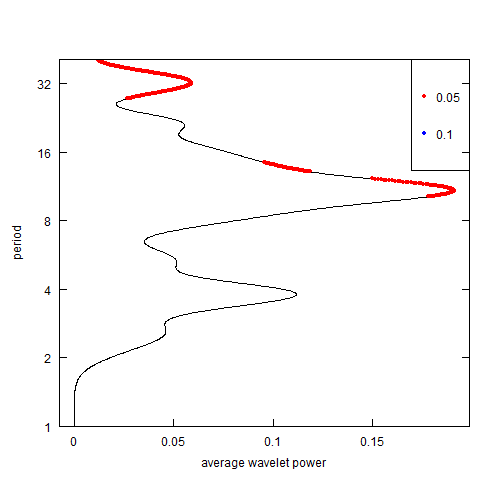 |
|  | | | |
|  | | | |
|  | | | |
| False Bay Seal 20 | | | |
| δ^13^C | | δ^15^N | |
| 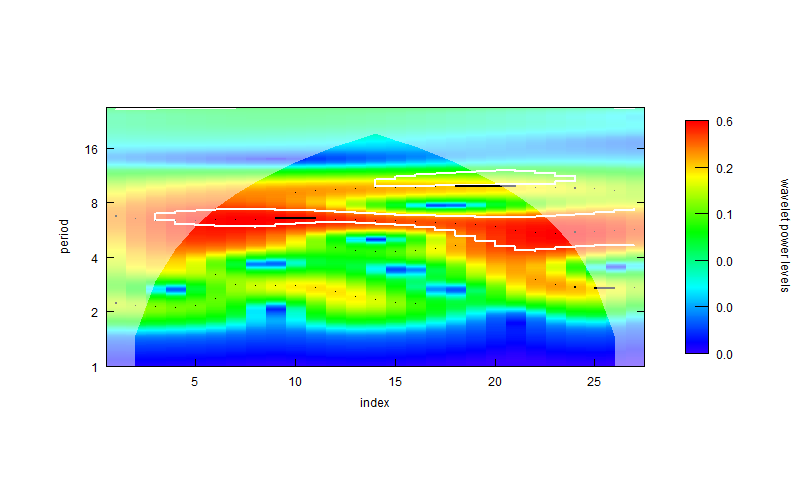 | 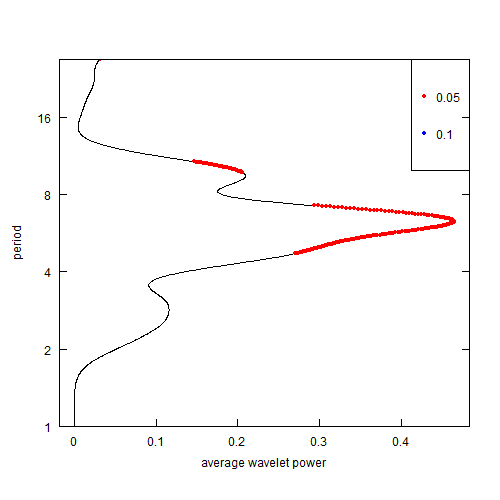 | 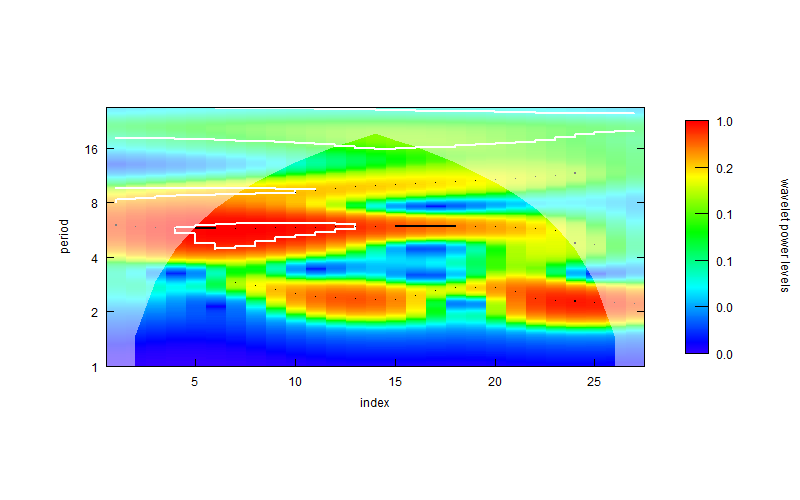 | 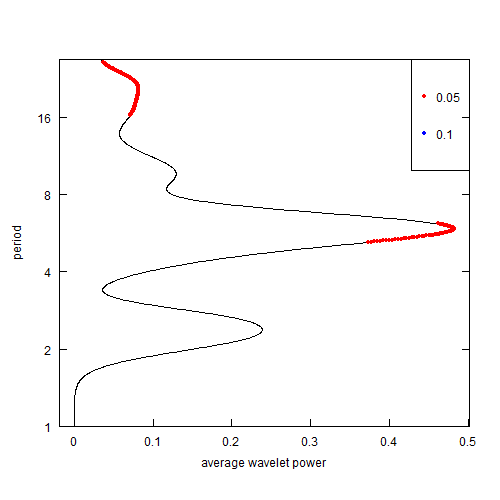 |
| False Bay Seal 21 | | | |
| δ^13^C | | δ^15^N | |
| 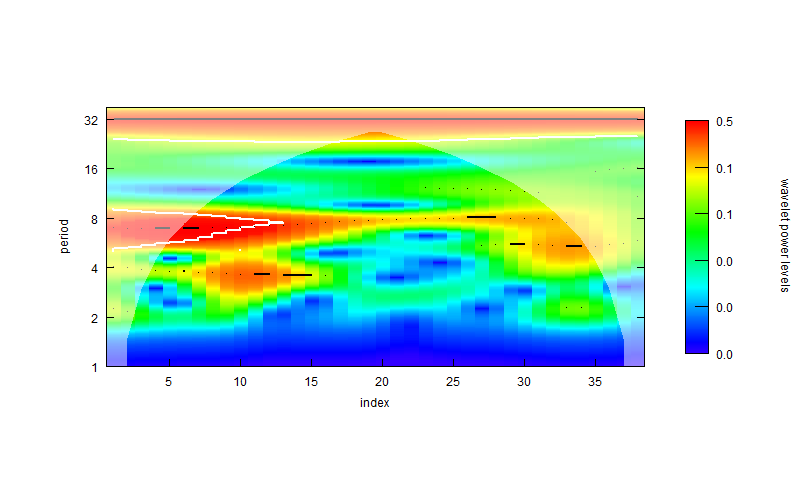 | 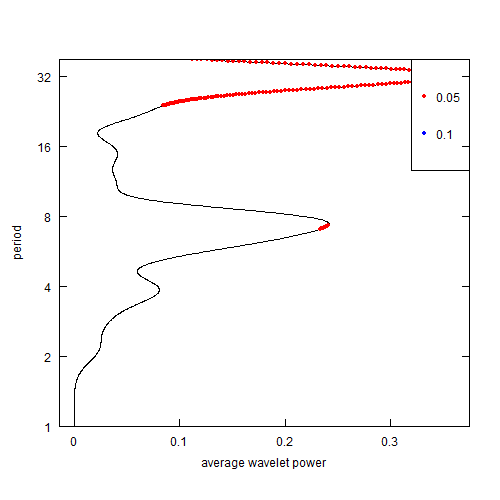 | 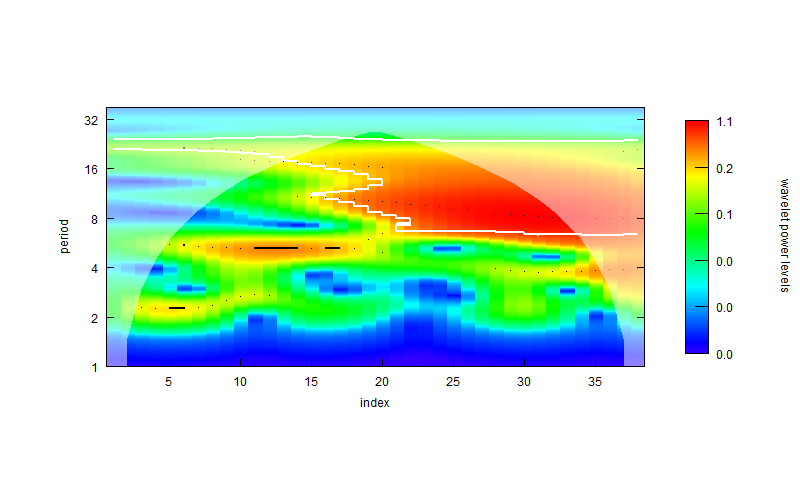 | 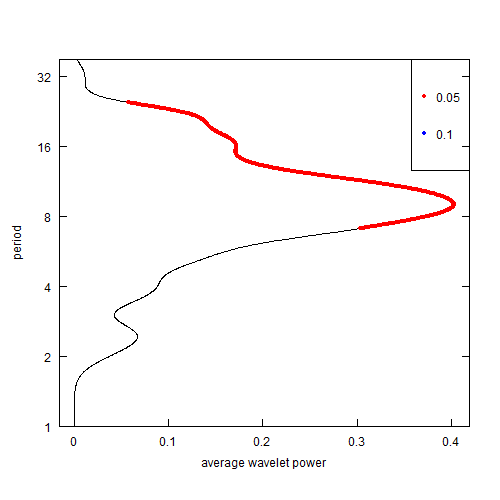 |

Fig A2 Results of the wavelet analysis conducted separately on δ^13^C and δ^15^N values for the whiskers of 21 female Cape fur seals from Kleinsee, Vondeling Island and False Bay
